# The BEACH Domain Is Critical for Blue Cheese Function in a Spatial and Epistatic Autophagy Hierarchy

**DOI:** 10.3389/fcell.2019.00129

**Published:** 2019-08-02

**Authors:** Joan Sim, Kathleen A. Osborne, Irene Argudo García, Artur S. Matysik, Rachel Kraut

**Affiliations:** ^1^School of Biological Sciences, Nanyang Technological University, Singapore, Singapore; ^2^Champalimaud Centre for the Unknown, Lisbon, Portugal; ^3^Singapore Centre on Environmental Life Sciences Engineering, Nanyang Technological University, Singapore, Singapore; ^4^Max Planck Institute of Molecular Cell Biology and Genetics, Dresden, Germany

**Keywords:** blue cheese, *Drosophila*, autophagy, BEACH domain, motor neuron, neurodegeneration

## Abstract

*Drosophila blue cheese* (*bchs*) encodes a BEACH domain adaptor protein that, like its human homolog ALFY, promotes clearance of aggregated proteins through its interaction with Atg5 and p62. *bchs* mutations lead to age-dependent accumulation of ubiquitinated inclusions and progressive neurodegeneration in the fly brain, but neither the influence of autophagy on *bchs*-related degeneration, nor *bchs’* placement in the autophagic hierarchy have been shown. We present epistatic evidence in a well-defined larval motor neuron paradigm that in *bchs* mutants, synaptic accumulation of ubiquitinated aggregates and neuronal death can be rescued by pharmacologically amplifying autophagic initiation. Further, pharmacological rescue requires at least one intact BEACH-containing isoform of the two identified in this study. Genetically augmenting a late step in autophagy, however, rescues even a strong mutation which retains only a third, non-BEACH containing isoform. Using living primary larval brain neurons, we elucidate the primary defect in *bchs* to be an excess of early autophagic compartments and a deficit in mature compartments. Conversely, rescuing the mutants by full-length Bchs over-expression induces mature compartment proliferation and rescues neuronal death. Surprisingly, only the longest Bchs isoform colocalizes well with autophagosomes, and shuttles between different vesicular locations depending on the type of autophagic impetus applied. Our results are consistent with Bchs promoting autophagic maturation, and the BEACH domain being required for this function.

**HIGHLIGHTS:**

The autophagic adaptor *blue cheese* is placed in an epistatic hierarchy, using pharmacological and genetic modulation of *bchs*- motor neuron degeneration. An intact BEACH isoform can promote autophagic proliferation, and in primary larval brain neurons Bchs shuttles to different components of the autophagy machinery, dependent on the stimulus.

## Introduction

A common pathological hallmark in many neurodegenerative disorders is the accumulation of intracellular toxic aggregates. Degradation of misfolded proteins and protein aggregates is mediated by two main intracellular systems, the ubiquitin-proteasome system (UPS) and autophagy (Glickman and Ciechanover, 2002; Levine and Klionsky, 2004). Both pathways are responsible for the recycling of different types of substrates depending on their solubility, half-life and composition, e.g., organelles versus misfolded proteins, type of substrate modification or the presence of specific degradation motifs (Nedelsky et al., 2008). Autophagy appears to function in part as a compensatory degradative pathway, because its activity increases when UPS is impaired (Iwata et al., 2005; Pandey et al., 2007). On the other hand, autophagic efficacy declines with ageing (Cuervo, 2008), acting as a possible mechanistic link to sporadic late-onset neurodegeneration.

During macroautophagy, elongation of the isolation membrane requires the E1-like activating enzyme Atg7 and E2-like conjugating enzyme Atg10 to bring Atg12 to Atg5 (an E3-like ligase) which then binds Atg16 to form the Atg12-Atg5-Atg16 multimeric complex (Ohsumi, 2001; Kuma et al., 2002). This E3-like ligase complex aids in the lipidation of Atg8 protein and dissociates from the membrane upon formation of the autophagosome (Kabeya et al., 2000; Hanada et al., 2007). Although the process of induction by signaling kinases to autophagosome formation and subsequent fusion with lysosomes has been extensively studied (Mizushima et al., 2002; Ganley, 2013), the receptors and adaptors that play a role in selective recognition of cargoes in specific cellular locations have been relatively unknown until recently (Lynch-Day and Klionsky, 2010). In mammals one of these adaptors, ALFY (autophagy-linked FYVE protein), scaffolds the machinery associated with isolation membrane elongation around sequestered protein aggregates by binding to the autophagy receptor, p62, through its PH-BEACH (Beige and Chediak–Higashi) domain, Atg5 through its WD40 repeats, and phosphatidylinositol 3-phosphate (PI3P)-containing autophagic membrane via its FYVE domain (Clausen et al., 2010; Filimonenko et al., 2010).

Loss of function mutations in *Drosophila blue cheese* (Bchs), the ortholog of ALFY, lead to age-dependent accumulation of ubiquitinated inclusions in adult brains, progressive degeneration throughout the nervous system and reduced adult longevity (Finley et al., 2003). A targeted genetic modifier screen in which lysosomal and autophagy candidate genes were able to modify a rough-eye phenotype induced by Bchs over-expression suggested that Bchs function may be involved in an autolysosomal trafficking pathway (Simonsen et al., 2007). A defect in size and anterograde transport of lysosomal compartments along motor neuron axons in *bchs* mutants supported this premise (Lim and Kraut, 2009). In both these studies, *bchs* mutants exhibited morphological features characteristic of atrophying neurons, such as axonal varicosities, ubiquitinated inclusions in the brain and disorganized microtubule bundles. Notably, the degeneration caused by loss of function for *bchs* was never tested for genetic modification by interference with autophagy.

A previous study has reported that ALFY, while not required for autophagy to occur, is associated with autophagic membranes and clears ubiquitinated aggregates from HeLa and neuronal cells (Simonsen et al., 2004; Filimonenko et al., 2010). However, no studies have examined in detail any changes in the autophagic machinery that may occur in *bchs*, or Bchs dynamic behavior with respect to autophagic components. Therefore, we set out to investigate the spatial localization of Bchs in relation to different steps along the autophagy-lysosomal pathway under various stresses and how these respond to *bchs* mutation.

We first present epistatic arguments, combining loss of function *bchs* alleles with genetic and pharmacological manipulations of autophagy, to show that Bchs occupies a specific position in an autophagy hierarchy that can be bypassed by late-acting Atg7, but not by early autophagic initiation. Further, we demonstrate that rescue of the degeneration by increasing autophagy requires at least one BEACH domain containing member of three newly identified isoforms, and that the full length protein strongly induces mature autophagic compartment formation, in keeping with earlier reported functions of BEACH domain proteins in vesiculation events.

## Results

### The *bchs* Locus Produces Three Isoforms That Are Differentially Localized With Autophagic Markers and Produce Different Phenotypes

**FIGURE 1:**
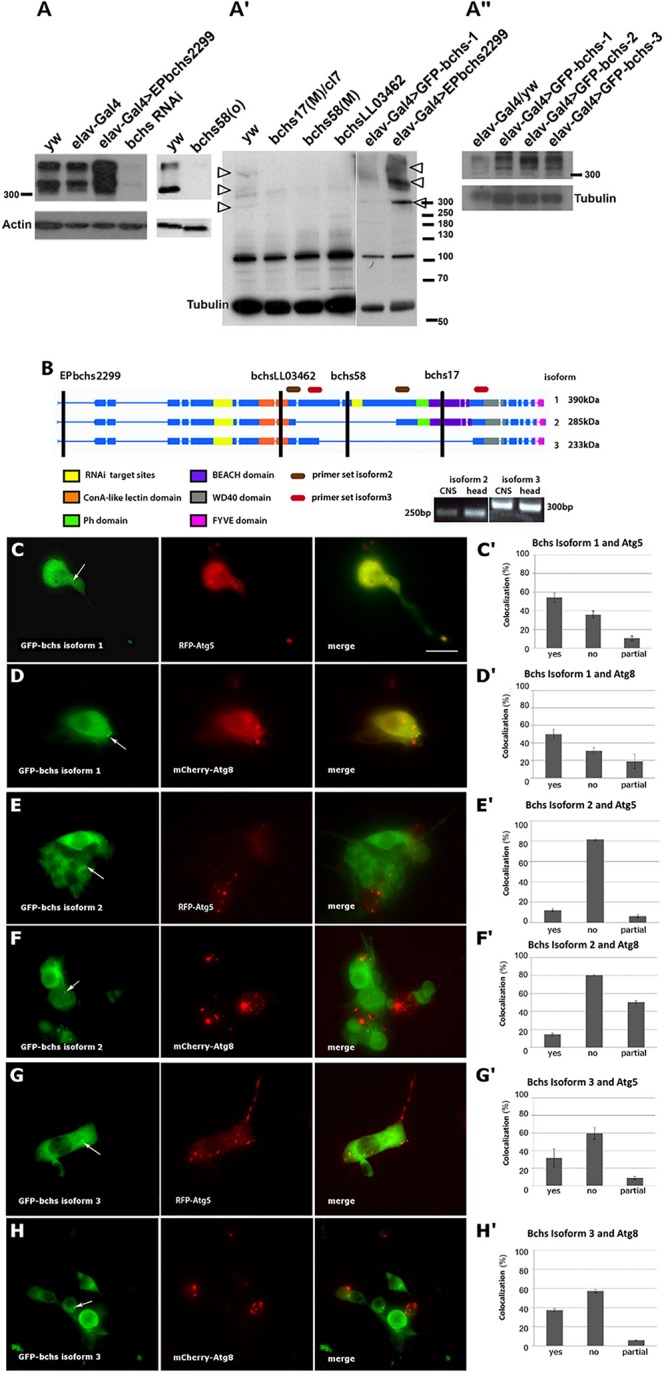
Blue cheese (Bchs) is alternatively spliced, and the isoforms colocalize to different extents with autophagosomes. **(A)** Left blot: Western of third instar larval brains from control wild type (*yw*) and elav-Gal4 alone, elav-Gal4-driven over-expression of *bchs* (elav-Gal4>EP*bchs*2299), and *bchs*RNAi. Right blot: adult heads, *yw* vs. *bchs58(O)*. **(A′)** Adult heads of *yw* vs. *bchs17(M)/Df(2L)cl7, bchs58(M)*, and *bchsLL03462* show loss of three bands >300 kDa (arrowheads). Right-most two lanes: shorter exposure of the identical blot, with elav-Gal4>GFP-bchs1 and elav-Gal4>EP*bchs*2299 showing increased expression of the same three separate bands >300 kDa (arrowheads) also present in *yw*, relative to non-specific band at ∼100 kDa. **(A^″^)** Adult heads of elav-Gal4 over *yw* control (left lane) vs. elav-Gal4 driven *bchs* isoforms 1, 2, and 3. Note that *bchs* isoform 2 is smaller than *bchs* isoform 1. Actin and tubulin were used as loading controls. **(B)** Schematic illustrating domains excluded from splice isoforms identified by RT-PCR from larval CNS and adult head. The positions of *EPbchs2299* insertion, *bchsLL03462, bchs58*, *bchs17* mutations are indicated by vertical black lines. Splice isoforms 1, 2, and 3 have expected sizes of 390, 285, and 233 kDa, respectively. However, note that the three products in part **A** (arrowheads) all appear to run >300 kDa; isoform 3 (right-most lane, **A^″^**) lacks the PH-BEACH domain and therefore may not be as well detected by the antibody, which was raised against the C-terminal third of Bchs, including the PH-BEACH domain (green, purple bands). Using the primer sets shown to detect splice isoform 2 (brown ovals) and splice isoform 3 (red ovals), 250 and 300 bp bands were detected, from which we deduced that splice isoform 1 is 2.8 kb larger than isoform 2 and 4.1 kb larger than isoform 3. **(C–H)** Live images and colocalization tally of elav-Gal4-driven **(C,C′)** GFP-bchs-1, the longest isoform, with RFP-Atg5 in primary larval neurons; **(D,D′)** GFP-bchs-1 with mCherry-Atg8a; **(E,E′)** GFP-bchs-2, which retains a BEACH domain, with RFP-Atg5; **(F,F′)** GFP-bchs-2 with mCherry-Atg8a; **(G,G′)** GFP-bchs-3, the non-BEACH isoform, with RFP-Atg5; **(H,H′)** GFP-bchs-3 with mCherry-Atg8a. Arrows point to vesicularly localized GFP-bchs isoforms. Contrast in GFP panels was enhanced to bring out visibility of the vesicles. Scalebar = 10 μm.

The existence of a single long isoform of Bchs has been reported, with smaller bands in Westerns being attributed to non-specific cross-reaction of the antibody (Khodosh et al., 2006; Simonsen et al., 2007). In our hands, Western blot analysis with a polyclonal antibody raised against the C-terminal 1008 amino acids of Bchs (Lim and Kraut, 2009) recognized three Bchs isoforms, all with apparent weights above 300 kDa, both in third instar larval brains and adult heads of *yw* control animals (Figures 1A,A′). A pan-neuronal Gal4 driver (elav-Gal4) driving expression via the gene insertion EP*bchs*2299 resulted in the over-expression of all three of these Bchs isoforms (Figure 1A; visible as separate bands in Figure 1A′, arrowheads), whereas driving *bchs* RNAi (Figure 1A) or mutation of *bchs* (Figure 1A′) resulted in loss of all three. The EMS alleles *beach17* and *beach58* [referred to here as *bchs58(O)*] were isolated by Khodosh et al. (2006) in the EP*bchs*2299 background. From these lines, we generated *bchs17(M)* and *bchs58(M)* by precise excision of the EP insertion. *bchs17(M)* introduces a stop codon at Trp2640, and is expected to disrupt isoforms 1 and 2 (Figure 1B). *bchsLL03462* is a strong allele resulting from an insertion into *bchs* of a splice acceptor site followed by stop codons into the 7th coding exon at aa 1229 preceding the BEACH domain (Flybase allele report FBti0124589). Our sequence analysis of *bchs58(O)*, which was characterized as a strong loss of function mutation, detects an insertion of 3 bases and a deletion of 17 bases, resulting in a frame shift and subsequent stop codon. Thus, the *bchs58* lesion is expected to remove only the longest Bchs isoform 1, but may produce a chimaeric protein that is not recognized by our antibody. However, the strong loss of all three bands in *bchs58(O)* and *bchs58(M)* (Figure 1A′), as well as the appearance of additional bands after overexpression of all three isoforms (Figures 1A′,A^″^), suggests that positive autoregulation by Bchs may be involved.

The three isoforms of Bchs (depicted in Figure 1B) were cloned as N-terminal GFP fusions and expressed in the nervous system via elav-Gal4 (Figure 1A^″^). Larval brains expressing each of the isoforms were dissociated into primary neuronal cultures (Figures 1C–H). Live imaging of these primary neurons in the background of transgenic RFP-Atg5 and mCherry-Atg8a expression to label early and late autophagic compartments showed that, in cases where GFP-Bchs can be detected in vesicles, only BEACH-containing isoform 1 (GFP-bchs-1) was predominantly coincident with autophagosomes (Figures 1C,D), whereas isoforms 2 and 3 had much less association with either Atg5 or Atg8 (Figures 1E–H), and indeed were often mutually exclusive in cells (this can be seen particularly well in Figures 1F,H). Autophagosomal association does not appear to depend on the BEACH domain, since BEACH domain-containing isoform 2 is less autophagosomal than isoform 3, which lacks the BEACH domain.

### Autophagic Modulation of Neurodegeneration in Loss of Function *bchs* Mutants Depends on the BEACH Domain

**FIGURE 2:**
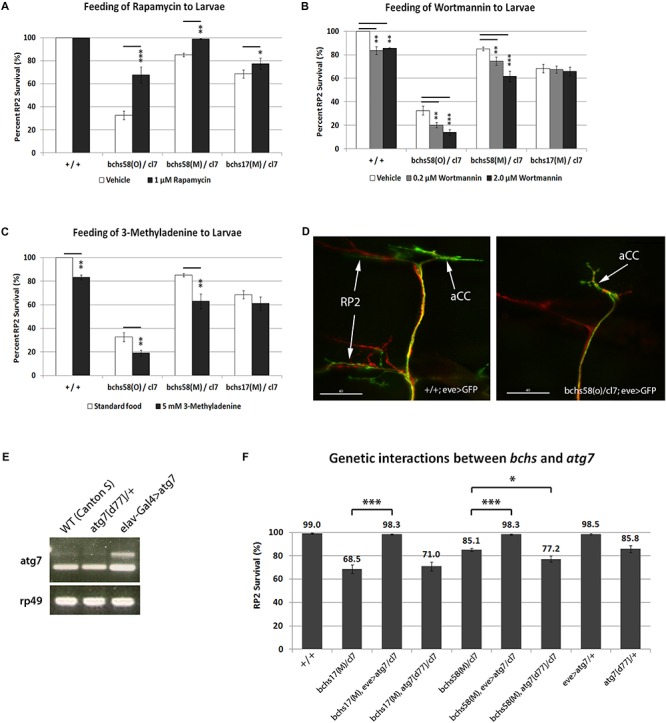
*bchs’* neurodegenerative phenotype is sensitive to autophagy-modulating drugs and genetic enhancement of autophagy. **(A–D)** Larvae of the indicated genotypes were fed on standard food containing either **(A)** rapamycin, **(B)** Wortmannin, or **(C)** 3-methyladenine. All third instar larval motor neurons were labeled as shown in the example in **(D)** with anti-Futsch (22C10; red), and co-labeled with anti-GFP to detect eve>GFP in aCC and RP2 motor neurons (green; arrows). RP2 is absent in the bchs mutant (right panel). Scalebar = 40 μM. Percent survival of RP2 motor neurons over ∼100 hemisegments was calculated for each treatment, and experiments were performed in triplicate. Chi-square test was used to determine statistical significance; ^*^p < 0.05, ^∗∗^*p* < 0.01, ^∗∗∗^*p* < 0.001. **(E)** Over-expression of *atg7* shown by RT-PCR on total RNA from adult heads of wild type (WT, CantonS), atg7[d77]/+ heterozygotes, and elav-Gal4>atg7, using primers specific for *atg7* mRNA and *rp49* rRNA as loading control. **(F)** Over-expression of *atg7* increases RP2 motor neuron survival in *bchs17(M)* and *bchs58(M)* (lanes 3 vs. 2 and 6 vs. 5). A single allelic deletion of *atg7* by itself (lane 9) gives a similar rate of RP2 death as *bchs58(M)* and mildly exacerbates it (lanes 5 vs. 7) but does not significantly change *bchs17(M)* (lanes 2 vs. 4). Experiments were performed in triplicate, and *N* > 100 (hemisegments) for each genotype. All genotypes include the driver/reporter combination eve>GFP in homozygosity. Error bars represent standard deviation, and Chi-square test was used to calculate statistical significance. ^*^p < 0.05, ^∗∗^*p* < 0.01, ^∗∗∗^*p* < 0.001.

A specific function of Bchs in aggrephagy was demonstrated by the observation that Bchs and its homolog ALFY interact physically with the autophagy machinery, and that both are able to reduce aggregates induced in human and *Drosophila* cells (Filimonenko et al., 2010). However, the loss of function neurodegenerative phenotype in the fly was not tested for interactions with either pharmacological or genetic modulators of autophagy, which should occur if *Drosophila* Bchs functions as an autophagic adaptor. Here, as in our earlier study (Lim and Kraut, 2009), the loss of larval aCC and RP2 motor neurons was used as a means of quantifying neurodegeneration in *bchs*. Using this method, we compared the extent of neurodegeneration in *bchs* alleles that differentially affect the three isoforms, and assessed the effects of autophagy-modulating drugs in these genetic backgrounds. aCC and RP2 motor neurons, which were shown to have a high frequency of TUNEL-labeling in the previous study, are specifically labeled in our assay by driving membrane-localized CD8-GFP with an *even-skipped* driver (this driver-reporter combination, used in Figure 2 and thereafter is referred to as eve>GFP). In combination with a genetic deficiency *Df(2L)cl7* (referred to as *cl7*), *bchs58(O)*, which retains the EP element of EPbchs2299, had lower motor neuron survival (∼32%) than either *bchs58(M)* (∼85%) or *bchs17(M)* (∼70%), the two alleles where the EP element was excised (Figure 2A). The phenotypic strength of the three alleles in the order *bchs58(O) > bchs17(M) > bchs58(M)* was thus established by this assay.

We next tested the effects of feeding the autophagy-modulating drugs rapamycin, 3-methyladenine, and Wortmannin on motor neuron death in larvae of the above allelic combinations. Larvae that were fed rapamycin, a well-established autophagy inducer (Rubinsztein and Nixon, 2010), grew more slowly (7–8 days vs. 3 days), and high concentrations (50 or 100 μM) resulted in widespread larval mortality before third instar. All treatments showed a change in the pattern of the mTOR effector phosphorylated S6 kinase (p-S6K) relative to controls in Westerns of larval brain (Supplementary Figure S1A). A comparison of mCherry-Atg8a in muscles of drug-fed larvae also showed increases or decreases in numbers of punctae (Supplementary Figure S1B), and rapamycin increased autophagosome formation (more Atg8 spots/cell) and flux (fewer p62 spots/cell) when applied to larval neurons in culture (Supplementary Figure S1D).

Feeding larvae rapamycin at 1 uM resulted in a significant amelioration of motor neuron death in all alleles over deficiency *cl7*, with *bchs58M* being rescued to nearly 100% survival (Figure 2A). Notably, however, the allele that putatively removes both BEACH domain isoforms, *bchs17(M)*, was rescued only marginally (from 70 to 78%). This suggests that the presumptive autophagic deficit in *bchs* mutants can be more effectively compensated by amplification of autophagy if a BEACH- isoform is present, as is the case in *bchs58*. Because rapamycin acts through inhibition of TORC1 and subsequent activation of the Atg1 complex (Ravikumar et al., 2004; Sarkar et al., 2008; Bové et al., 2011), an epistatic interpretation of this result would be that complete rescue requires Bchs with an intact BEACH domain, downstream of Atg1 in the autophagy hierarchy. It also identifies BEACH-containing isoforms of Bchs as the primary participants in autophagy.

Wortmannin and 3-methyladenine (3-MA) are widely used broad-spectrum phosphatidylinositol 3-kinase (PI3K) inhibitors that act mainly via the suppression of class III PI3K activity to inhibit the nucleation of phagophores through PI3P production (Seglen and Gordon, 1982; Powis et al., 1994). 0.2 or 2 μM Wortmannin significantly reduced motor neuron survival in wild-type control, *bchs58(O)/cl7* and *bchs58(M)/cl7*, but did not exacerbate *bchs17(M)/cl7* (Figure 2B), the genetic background that lacks the two BEACH-containing isoforms. Similarly, suppression of autophagy by 3-MA caused motor neuron death in wild-type (phenocopying *bchs* motor neuron loss, a typical example of which is shown in Figure 2D) and exacerbated *bchs58(O)/cl7* and *bchs58(M)/cl7*, but did not significantly exacerbate *bchs17(M)/cl7* (Figure 2C). These data again point to the BEACH domain isoforms being responsible for the residual autophagic capacity that appears to be present in the rescuable *bchs58* alleles.

### Activation of a Late-Stage Autophagy Step by *atg7* Rescues the *bchs* Degenerative Phenotype

There is evidence that ALFY interacts physically with the autophagy machinery, but a *bona fide* genetic interaction between a loss of function *bchs* phenotype and autophagy has not been tested. Therefore, we examined whether manipulation of autophagy genes could modify *bchs* motor neuron death. Atg7, an E1-like ubiquitin activating enzyme that is involved in the conjugation of phosphatidylethanolamine (PE) to Atg8, and formation of the Atg5-Atg12 conjugate, has been shown to suppress the accumulation of ubiquitinated aggregates, thereby contributing to *Drosophila* adult longevity (Juhasz et al., 2007; Geng and Klionsky, 2008). Adult heads of *Atg7[EY10058]*, which has upstream activating sequences (UAS) inserted before the *atg7* transcriptional start site, driven with elav-Gal4 (elav-Gal4>atg7 in Figure 2E), showed an increase in *atg7* transcripts by reverse-transcription (RT)-PCR (Figure 2E, lane 3), and an increase in p-S6K compared to control (Supplementary Figure S1C). Therefore, we used this line to over-express Atg7 in the presence or absence of Bchs.

Strikingly, over-expression of Atg7 via eve-Gal4 (eve>atg7 in Figure 2F) rescued motor neuron survival to almost 100% in both the strong allele *bchs17(M)/cl7* and the hypomorph *bchs58(M)/cl7* (Figure 2F, lanes 3 and 6). Notably, this genetic augmentation of a later-occurring autophagy step via Atg7 was more effective than rapamycin feeding at rescuing *bchs17(M)*, which only mildly improved *bchs17(M)/cl7* degeneration (Figure 2A). *bchs58(M)/cl7* showed a similar percentage of neuronal death as the loss of function allele *atg7[d77]/*+ (85.1% vs. 85.8%; Figure 2F, lanes 5 and 9) and combining them resulted in a further reduction of neuronal survival to 77.2% (Figure 2F, lane 7). In contrast, combination with *atg7* did not significantly exacerbate the phenotype of *bchs17(M)/cl7* (Figure 2F, lanes 2 vs. 4). Another *atg7* deletion *atg7[d14]* was synthetic lethal when recombined with *cl7*, making it difficult to examine the effect of *atg7*- homozygosity on *bchs*.

Atg1 activity has previously been shown to rescue phenotypes that result from autophagic deficit by overexpression of a transgene (Scott et al., 2007). We attempted this in the background of *bchs* alleles, using two different available constructs (UAS-Atg1^*GS10797*^, and UAS-Atg1(6A), a gift of T. Neufeld), but both of these resulted in nearly complete motor neuron death when expressed in the neurons to be assayed, alone and in combination with *bchs* alleles, and could therefore not be tested for rescue.

In summary, *bchs* alleles with or without BEACH products can be rescued substantially by enhancing a late autophagic step via Atg7 over-expression. Conversely, *bchs17(M)*, which removes both BEACH-containing isoforms, cannot be further exacerbated by disrupting autophagic activity via *Atg7* knockout. Both of these observations point to *atg7* acting in one functional pathway downstream of *bchs*, and its ability to override loss of BEACH isoforms.

### Accumulation of Ubiquitinated Aggregates in *bchs* Motor Neuron Termini Accompanies Degeneration and Is Abolished by Augmenting Autophagy

Previous reports have demonstrated the accumulation of insoluble ubiquitinated protein aggregates in aged *bchs* adult heads and in photoreceptor axons of Bchs over-expressing late pupae (Finley et al., 2003; Simonsen et al., 2007). Larval motor neurons are ideally suited to examine the subcellular localization of such aggregates due to their clearly identifiable anatomy. Therefore, we investigated whether ubiquitinated aggregates accumulate in *bchs* motor neurons and where this occurs.

**FIGURE 3:**
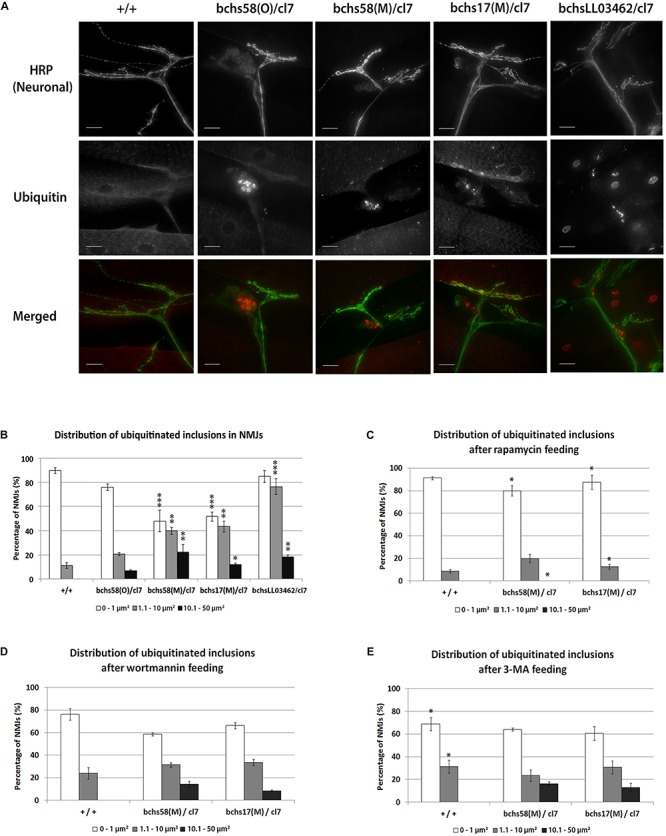
Ubiquitinated aggregates accumulate in neuronal termini of *bchs* larval neuromuscular junctions and can be reduced by rapamycin feeding. **(A)** Third instar larvae were dissected and immunostained for anti-HRP (a pan-neuronal marker) and anti-poly-ubiquitin. Scale-bar = 20 μm. **(B)** Percentages of neuromuscular junctions with ubiquitinated inclusions in each of three size groups (see Materials and Methods) were calculated for the different genotypes. Experiments were performed in duplicate, and error bars represent S.E.M. Chi-square test was used to determine pairwise statistical significance between frequency of occurrence of a given aggregate class in *bchs* vs. the same class in the wild-type control. ^*^p < 0.05, ^∗∗^*p* < 0.01, ^∗∗∗^*p* < 0.001. Larvae of the indicated genotypes were fed on **(C)** rapamycin, **(D)** Wortmannin, or **(E)** 3-methyladenine (see Materials and Methods) before repeating the same procedures as in **(B)** for analysis. Stars in **(A)** show significance of differences between classes in each of the genotypes and wild-type (+/+). Stars in **(C)** and **(E)** show significance for pairwise comparisons of numbers of ubiquitinated inclusions in the same genotype in **(B)** without drug.

Aggregates appeared prominently in the dorsal-most synaptic arbors of motor neurons in third instar larval body walls of *bchs* animals (Figure 3A). There was no obvious accumulation of ubiquitinated conjugates in motor neuron axons and cell bodies in *bchs* mutants (data not shown). For quantification, aggregate area sizes were categorized into three groups: 0–1 μm^2^ (small), 1.1–10 μm^2^ (medium), and 10.1–50 μm^2^ (large). While the smallest ubiquitinated aggregates were nearly always found in wild type termini, the frequency of medium- and large-sized aggregates was much higher in *bchs* mutants. The allele *bchsLL03462/cl7* had the highest occurrence of medium aggregates, whereas *bchs58(O)/cl7* had fewer overall (Figure 3B). Although this result was unexpected based on the severity of the alleles, it may be related to the possibly neomorphic nature of the *58(O)* allele.

We next investigated whether autophagy-modulating drugs affected the distribution of aggregates in termini, and whether this correlated with motor neuron viability. *bchs58(M)/cl7* and *bchs17(M)/cl7* fed 1 μM rapamycin showed significantly lower frequencies of medium and large ubiquitinated aggregates, while small aggregates increased significantly (Figure 3C; significance was calculated in comparison to frequency of a given aggregate size in the corresponding genotype without drug in Figure 3A).

In contrast to rapamycin feeding, Wortmannin and 3-MA did not affect the distribution of ubiquitinated aggregates in *bchs* mutants (Figures 3D,E, compare to B). However, 3-MA feeding caused a significant shift toward the formation of medium (but absence of large) ubiquitinated aggregates in wild-type larvae (Figure 3E).

### Early Autophagosomes Increase in *bchs* Primary Neurons and Bchs Expression Drives Progression to Late Autophagy

**FIGURE 4:**
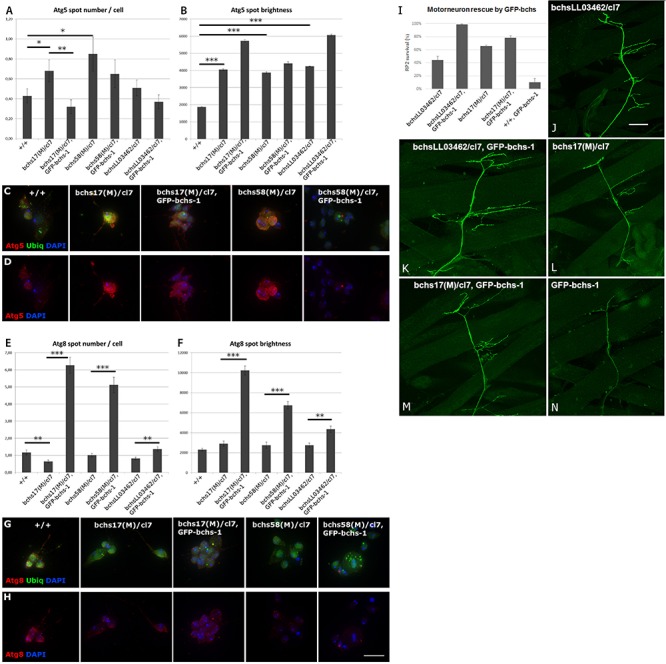
Full-length Bchs drives an increase in transition of early Atg5-positive vesicles to Atg8-carrying autophagosomes, and full-length Bchs rescues *bchs* neuronal loss, but kills motor neurons in a wild-type background. **(A,B)** Early autophagic Atg5-positive vesicle number and brightness (fluorescence intensity per pixel within a spot) in primary larval neurons, with examples of neurons of individual genotypes **(C,D)**, also labeled with anti-poly-ubiquitin (green) and DAPI (blue) to show cell locations. ^*^p < 0.05, ^∗∗^*p* < 0.01, ^∗∗∗^*p* < 0.001. **(E,F)** Late autophagic Atg8-positive vesicle number and brightness in the same genotypes as in **(A,B)**; examples shown in **(G,H)**. Atg5/Atg8 and DAPI are shown separately to make changes in abundance of the respective marker more visible. Scalebar = 10 μm. **(I–N)** RP2 motor neuron survival after GFP-bchs-1 expression via eve-Gal4 in *bchs* mutant backgrounds *bchsLL03462/cl7*, *bchs17(M)/cl7*, or in the control background (+*/*+). **(J–N)** Images of representative neuromuscular junctions in the larval body wall labeled with eve>GFP in the same *bchs* backgrounds, with or without GFP-bchs-1. Scalebar = 20 μm.

Since the observed accumulation of ubiquitinated aggregates in neuronal termini of *bchs* neuromuscular junctions suggests a blockage in the autophagy pathway, it was of interest to investigate where this blockage might be taking place. Because primary neurons prepared from third instar larval brains could be analyzed in much larger numbers than larval motor neurons, we used these to assess the number per cell and brightness of Atg5- and Atg8-positive compartments (Figures 4A–H). Atg5-positive compartments increased significantly in number and/or brightness in all *bchs* allelic combinations (Figures 4A,B), whereas Atg8-positive compartments were reduced, but only significantly in *bchs17(M)* mutants (Figures 4E,F) (cell images of additional *bchs* allelic combinations shown in Supplementary Figure S2). Ubiquitin-labeled inclusions were visible in all preparations, but appeared more prominent and vesicular rather than predominantly nuclear and cytoplasmic, in the neurons that were rescued by GFP-bchs-1 (Figures 4C,G).

Interestingly, expression of GFP-bchs-1 in *bchs* mutant backgrounds resulted in an increase in brightness of Atg5 in vesicles (Figure 4B, sample 3), but a reversal of the increase in Atg5 compartment number (Figure 4A, sample 3). Even more strikingly, GFP-bchs-1 expression also caused a very strong increase in both Atg8 compartment number and intensity over wild type (Figures 4E,F, sample 3; Figure 4H, panels 3, 5). Full multi-cell comparisons of Atg5 and Atg8 expression in the different genotypes are shown as montages in Supplementary Figures S3–S6. We also noted that both Atg5 and Atg8 accumulated detectably in the neurites of *bchs17(M)/cl7* compared to wild-type in neurons that had been allowed to differentiate further by aging for 4 additional days in culture (Supplementary Figures S4B, S6B). These neurons also showed notably more prominent ubiquitinated aggregates than the younger preparations (see enlarged insets, green in Supplementary Figures S4B, S6B).

In general, our findings with respect to Atg8 differ from those of Filimonenko et al. (2010), who reported no effect on starvation-induced LC3 expression in ALFY knockdowns. This discrepancy may be due to differences in methods of quantification, cell type, or presence vs. absence of autophagy induction. Surprisingly, the loss of *bchs* did not influence the localization of Huntingtin (Htt) Q93 ubiquitin aggregates with either Atg5 or Atg8 (Supplementary Figure S7), indicating that Bchs does not have an explicit role in guiding these aggregates to the nascent or fully formed autophagosome.

In order to discern whether *bchs*’ effects on autophagic compartments were due to changes in flux, we treated control and *bchs*RNAi-treated primary neurons expressing mCherry-Atg8 with chloroquine in order to block auto-lysosomal degradation (Supplementary Figures S8A–F). Chloroquine reduced mCherry-Atg8 size and intensity significantly over controls, and this was strongly reversed by the presence of *bchs*RNAi. Additionally, we tested *bchs*’ effects on another marker of autophagosomal maturation, Syntaxin 17 (Syx17), a vesicular synaptosome-associated protein receptor (SNARE) necessary for HOPS-mediated lysosomal-autophagosomal fusion (Takáts et al., 2014; Supplementary Figures S8G–L). Changes in expression and localization of Syx17 were evident: *bchs17(M)/cl7* appeared to increase the size of Syx17 compartments, while *bchs58(M)/cl7* neurons more often had smaller, more tightly localized Syx17 (Supplementary Figure S8L). Overexpression of GFP-bchs-1 notably resulted in large areas of Syx17 throughout the cell body and in the nucleus (Supplementary Figures S8K,L).

The functionality of Bchs-GFP isoform 1 —the most clearly autophagosome-associated isoform (see Figure 1)—which induced Atg8 compartment formation in the foregoing experiment (Figure 4), was assayed for its ability to rescue the motor neuron loss of different *bchs* loss of function alleles. To test this, GFP-bchs-1 was recombined onto the *cl7* deficiency chromosome, and crossed into the background of different *bchs* combinations. *bchsLL03462* by itself gave only ∼40% motor neuron survival, but was rescued by the transgene GFP-bchs-1 to ∼100% survival (Figure 4I). The ∼68% survival seen in *bchs17(M)* was mildly rescued to ∼80%, while *bchs58(M)* was not rescued (not shown). Expression of GFP-bchs-1 by itself resulted in extensive motor neuron loss, suggesting that particular levels or a balance of the three isoforms may be important.

We conclude from the above set of experiments that *bchs* neurons experience an autophagic block leading to an excess of early Atg5 compartments, which can be overcome by expression of the full-length Bchs isoform. Indeed, Bchs isoform1 appears to induce super-normal numbers and intensities of Atg8 vesicles (Figures 4E,F), as well as increasing the extent of Syx17 domains, which together reflect the mature autophagosome (Supplementary Figure S8). This indicates a likely function of the full-length Bchs protein in maturation of autophagosomal compartments.

### Bchs Colocalizes Preferentially With Atg5 During Selective Autophagy and With Atg8 During Non-selective Autophagy

**FIGURE 5:**
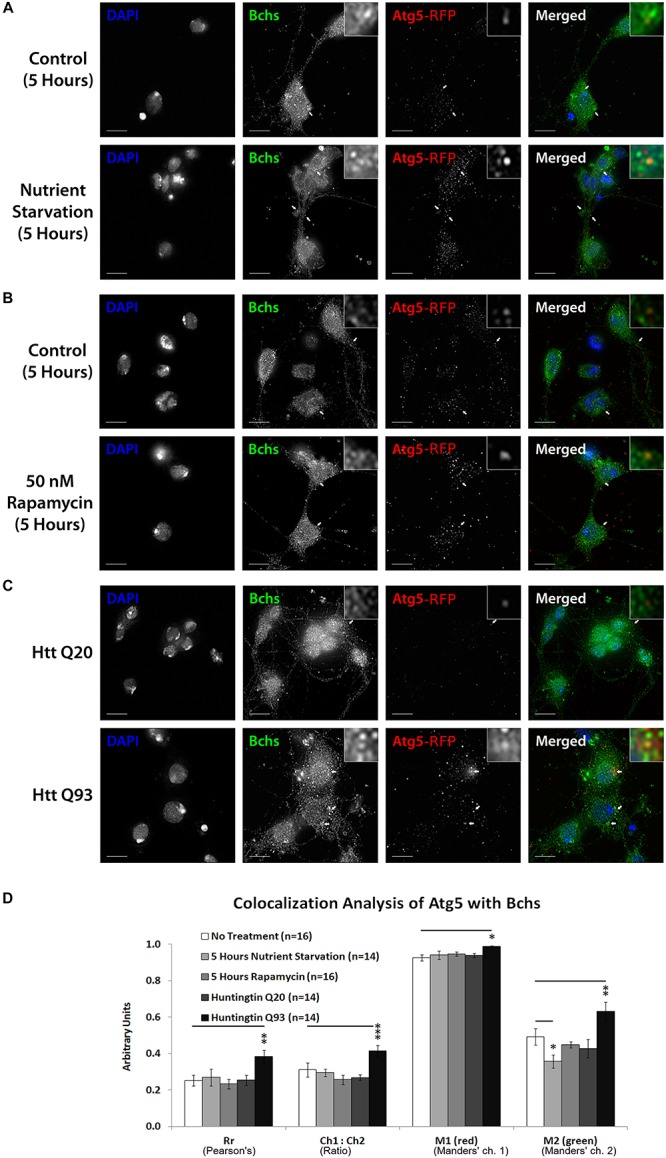
Bchs associates with RFP-Atg5 marked compartments during aggrephagy and dissociates from them during starvation-induced autophagy. Third instar larval primary neurons were immunostained for endogenous Bchs and transgenic RFP-Atg5 using anti-Bchs and anti-DsRed antibodies after different conditions of autophagy induction: **(A)** five hours of nutrient starvation by incubation with serum-free HL-3 buffer, **(B)** five hours of incubation with 50 nM rapamycin in complete medium, and **(C)** Htt normal (Q20) and expanded (Q93) polyQ expression. Scalebar = 5 μm. Arrowheads indicate regions of close association between Bchs and RFP-Atg5. The top-right inset shows a 10× magnification of one of the indicated regions. **(D)** Colocalization analysis was performed using ImageJ to obtain Rr (Pearson’s correlation coefficient), Ch1:Ch2 ratio (red:green pixels ratio), thresholded M1 (Manders’ colocalization coefficient for Channel 1) and M2 (Manders’ colocalization coefficient for Channel 2). Error bars represent standard error of the mean for n = number of single-slice images. Unpaired Student’s *t*-test was used for the statistical comparison between treatment and non-treatment groups. ^*^p < 0.05, ^∗∗^*p* < 0.01, and ^∗∗∗^*p* < 0.001.

**FIGURE 6:**
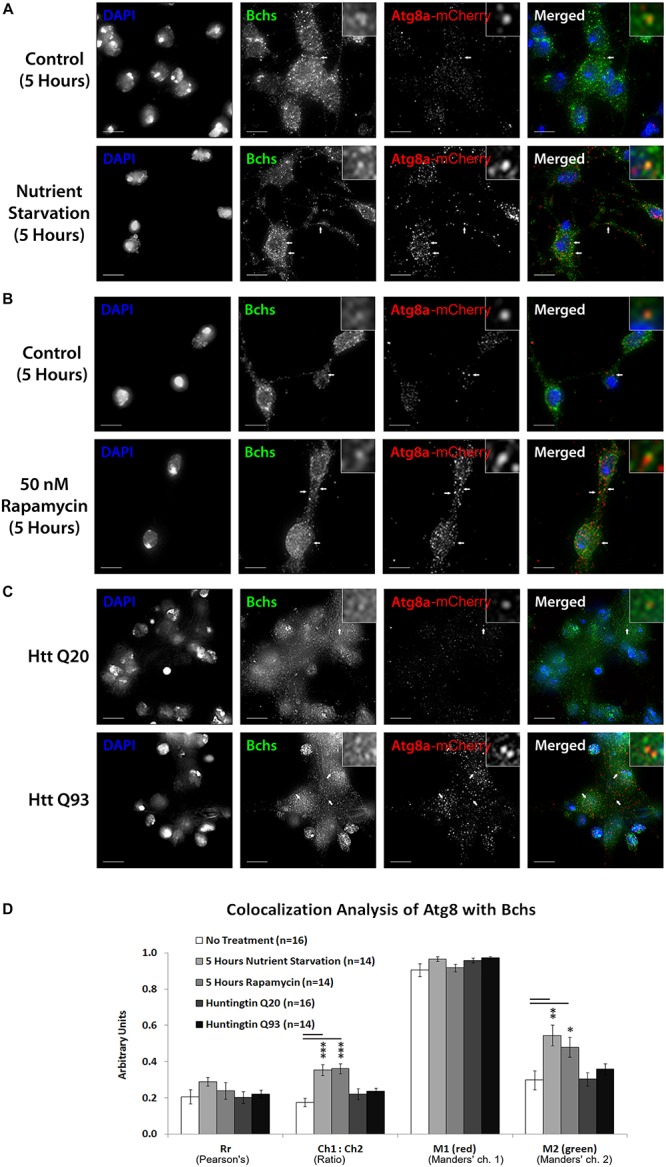
Nutrient starvation and rapamycin treatment gave a significant increase in the relative quantity of mCherry-Atg8a to Bchs (expressed by the Ch1:Ch2 value), likely explained by an increase in vesicular localization of the transgenically expressed tagged mCherry-Atg8a protein upon autophagy induction **(A,B,D)**. The amount of Bchs colocalizing with this mCherry-Atg8a (M2) also increased significantly **(D)**. In contrast, Htt Q93 expression did not increase M2 **(C,D)**, as it had with RFP-Atg5 (compare Figure 5D). These results show that Bchs increases its localization with mCherry-Atg8a-tagged compartments upon general (starvation-induced) autophagy, but not after aggregate induction.

After autophagy stimulation by nutrient starvation, rapamycin treatment and Htt polyQ93 expression, it was observed generally that the size and number of individual Bchs, Atg5 and Atg8 compartments increased compared to non-treated controls (Figures 5, 6). However, Bchs was homogeneously distributed throughout the primary neuron and colocalized only ∼20–25% with either marker, RFP-Atg5 or mCherry-Atg8a, under basal and induced autophagy (Figures 5, 6), which is surprising in light of earlier findings with Alfy (Simonsen et al., 2004). We also note that in cases of overlap between Bchs and the respective markers, the punctae do not completely colocalize with each other (see merged images in Figures 5, 6), suggesting their localization on different compartments or sub-compartments.

The colocalization of endogenous Bchs with transgenically expressed compartmental markers in response to different stimuli was analyzed using Intensity Correlation Analysis (ICA) (Li et al., 2004; Schneider et al., 2012). The Manders’ colocalization coefficient for the green channel (M2) (i.e., the proportion of the intensity in Bchs-positive (green) pixels overlapping with that in RFP-Atg5-positive (red) pixels), decreased during nutrient starvation, but not under rapamycin treatment (Figures 5A,B,D), indicating that dissociation from Atg5 compartments may be a specific response to starvation as opposed to general autophagic induction. In contrast, the expression of Htt Q93 (but not Q20) led to a significant increase in Rr, the Pearson’s correlation coefficient, M1 (Manders’ coefficient for the red channel) and M2 values with RFP-Atg5 (Figures 5C,D). The ratio of RFP-Atg5 intensity to endogenous Bchs intensity, expressed by Ch1:Ch2, also increased, although the transgenic RFP-tagged protein is expected to be expressed at constant levels. This may be due to the clearly visible increase in punctate localization of this marker upon autophagic induction, which would be expected to be enhanced by the colocalization algorithm during background subtraction. These results together suggest that Bchs may associate preferentially with early autophagosomal Atg5 compartments during aggrephagy, and at least partially dissociate from them during starvation-induced autophagy, moving toward an Atg8-associated compartment.

### Selective Autophagy Lowers Bchs Colocalization With Rab11-GFP

**FIGURE 7:**
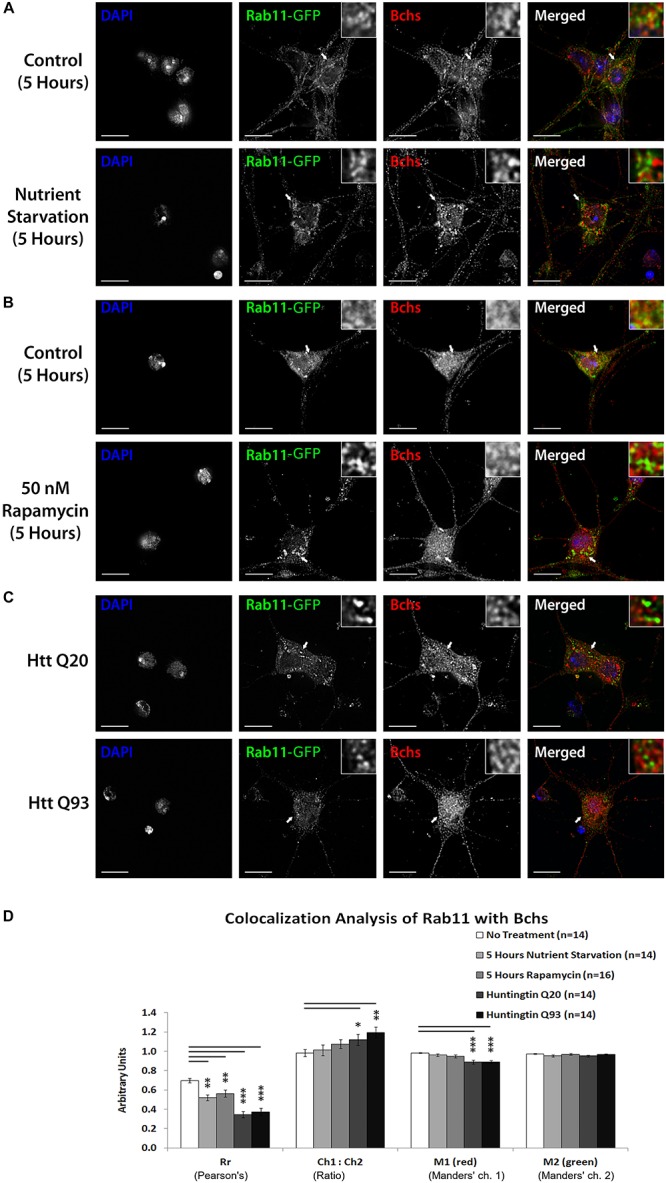
Bchs dissociates more from Rab11-GFP during selective autophagy than non-selective autophagy. Third instar larval primary neurons were immunostained for endogenous Bchs and transgenic Rab11-GFP using anti-Bchs and anti-GFP antibodies after different conditions of autophagy induction: **(A)** five hours of nutrient starvation by incubation with serum-free HL-3 buffer, **(B)** five hours of incubation with 50 nM rapamycin in complete medium, and **(C)** Htt normal (Q20) and expanded (Q93) polyQ expression. The top-right inset shows a 5× magnification of one of the indicated regions. Scalebar = 5 μm. **(D)** Colocalization analysis was performed using ImageJ to obtain Rr (Pearson’s correlation coefficient), Ch1:Ch2 ratio (red:green pixels ratio), M1 and M2 (Manders’ colocalization coefficients for channels 1 and 2). Error bars represent standard error of the mean for n = number of single-slice images. Unpaired Student’s *t*-test was used for the statistical comparison between treatment and non-treatment groups. ^*^p < 0.05, ^∗∗^*p* < 0.01, and ^∗∗∗^*p* < 0.001.

Rab11 localizes onto recycling endosomes and is required for early endocytic membrane trafficking and recycling (Ullrich et al., 1996; Wilcke et al., 2000). Bchs colocalizes partially with Rab11-GFP in *Drosophila* embryonic motor neurons and Rab11 antagonizes Bchs function in synaptogenesis (Khodosh et al., 2006; Lim and Kraut, 2009). To look at how this interaction is affected by autophagy, the spatial relationship of Bchs with Rab11-GFP was investigated in primary neurons in response to autophagy induction by different conditions. After autophagy induction by all three methods – nutrient starvation, rapamycin treatment or Htt polyQ expression – there was a reduction in colocalization between Bchs and Rab11-GFP as measured by both the Pearson’s correlation coefficient (Rr) and M1 (the proportion of Bchs intensity overlapping with Rab11-GFP), with Htt polyQ giving the strongest reduction (Figures 7A–D). These data show that Bchs associates less with Rab11 recycling compartments during the induction of selective (aggregate-induced) than under non-selective (starvation or rapamycin-induced) autophagy. However, the number of rab11-carrying compartments appeared not to be affected by autophagy induction, tested by using a YFP insertion in the rab11 gene [a gift of M. Brankatschk and S. Eaton (Dunst et al., 2015)] in primary neurons treated with rapamycin (not shown).

### Live Imaging of GFP-Bchs With RFP-Atg5 or mCherry-Atg8a During Autophagy Induction Confirms Stimulus-Dependent Relocalization of Bchs

In order to study the dynamics of Bchs interaction with autophagic vesicles, primary neurons cultured from third instar larval brains expressing GFP-bchs-1 and RFP-Atg5 or mCherry-Atg8a were imaged live. GFP-bchs-1 punctae are localized adjacent to RFP-Atg5 punctae occasionally under basal autophagy (Supplementary Figure S9 and Supplementary Movie S1). After nutrient starvation, GFP-Bchs and RFP-Atg5 appeared more distinct (Supplementary Figure S9B and Supplementary Movie S2), in agreement with the reduction in colocalization seen in fixed preparations (Figure 5D). RFP-Atg5 also re-locates nearer to the nuclear membrane after 40 min of starvation (Supplementary Figure S9B). These structures may correspond to the phagophore assembly sites, which are thought to form from omegasomes on the ER (Hayashi-Nishino et al., 2009; Ylä-Anttila et al., 2009).

After transfection of Htt Q15, occasional GFP-Bchs punctae adjacent to RFP-Atg5 punctae were observed (Supplementary Figure S9C and Supplementary Movie S3), similar to basal conditions. Htt Q128 transfection resulted in observable colocalization of GFP-bchs-1 and RFP-Atg5 in live neurons (Supplementary Figure S9D and Supplementary Movie S4). GFP-bchs-1 in these experiments was distributed mainly homogeneously throughout the cytosol, similarly to endogenous Bchs.

Under basal autophagy conditions, some mCherry-Atg8a punctae were adjacent to GFP-Bchs punctae (Supplementary Figure S9E and Supplementary Movie S5). After 2 h of nutrient starvation, however, there was a distinct enlargement of mCherry-Atg8a autophagosomes (Supplementary Figure S9F and Supplementary Movie S6). These doughnut-shaped punctae progressed to a bean-shaped forms, resembling the cross-section of vesicular compartments (visible in Supplementary Figure S9F).

When Htt Q15 or Q128 were expressed, there was little observable coincidence between mCherry-Atg8a and GFP-Bchs in live cells (Supplementary Figures S9G,H and Supplementary Movies S7, S8), similar to basal conditions and corroborating the colocalization analysis (Figure 6D). mCherry-Atg8a accumulated in a prominent focal swelling along the axon (left arrow in Supplementary Figure S9H), and an mCherry-Atg8a streak can be seen moving retrograde toward the focal swelling, but does not exit from it toward the cell soma, seeming to indicate a blockage of autophagosomal transport.

## Discussion

Physical interactions of the proposed aggrephagy adaptor and p62-binding BEACH domain protein (Nezis et al., 2008; Clausen et al., 2010) Bchs with other parts of the autophagic machinery have been demonstrated in biochemical studies. However, a convincing demonstration of how autophagic manipulation might affect neurodegeneration in a *bchs* mutant phenotype has never been provided. This is important for two reasons: first, it is critical to examine effects on the intrinsic neuronal degeneration caused by loss of function of the gene, rather than relying solely on ectopic expression of aggregating proteins and over-expression of the Bchs product, or parts thereof (Finley et al., 2003; Simonsen et al., 2007; Clausen et al., 2010; Filimonenko et al., 2010). Second, modulating different steps in the autophagic pathway in the presence of mild vs. severe loss-of-function *bchs* alleles allows the placement of *bchs* in a hierarchical framework, thus giving clues to its possible function.

In this study, we carry out epistasis analyses by demonstrating that the degree of motor neuron loss in *bchs*, described in an earlier publication as representing a quantitative measure of degeneration, can be rescued by genetic and pharmacological autophagy induction, but only in *bchs* alleles that retain at least one of the three newly discovered BEACH-containing protein isoforms. Indeed, we observe that these isoforms are not equally or even predominantly associated with autophagosomes in primary larval brain neurons, and in fact the two shorter isoforms are often seen to be mutually exclusive with Atg5 and Atg8 expression in individual neurons (see Figure 1). Our autophagic interaction results suggest that the BEACH domain is essential for autophagic enhancement to be able to rescue neurons from degeneration. This was surprising, since Filimonenko et al. found that the WD40/FYVE region alone was responsible for binding Atg5 and could by itself mediate aggregate clearance (Filimonenko et al., 2010). One interpretation of this apparent contradiction is that aggregate non-clearance by itself may not be the sole or even primary cause of degeneration. Indeed, the most strongly degenerative loss of function allele, *bchs58(O)*, did not have the most severe aggregate accumulation.

Autophagy inhibiting drugs not only exacerbated neuronal death in the weaker *bchs* mutants but, importantly, also phenocopied *bchs*, showing that autophagy inhibition alone induces a similar extent of motor neuron death as is seen in *bchs* larvae. However, strong loss of function of *bchs* apparently impairs autophagy to such an extent that it is refractory to further exacerbation by these drugs. Consistent with this, strong *bchs* loss of function is only marginally significantly rescued by rapamycin, which triggers Atg1 activity, leading to the conclusion that Bchs functions downstream of Atg1 (Mizushima, 2010).

**FIGURE 8:**
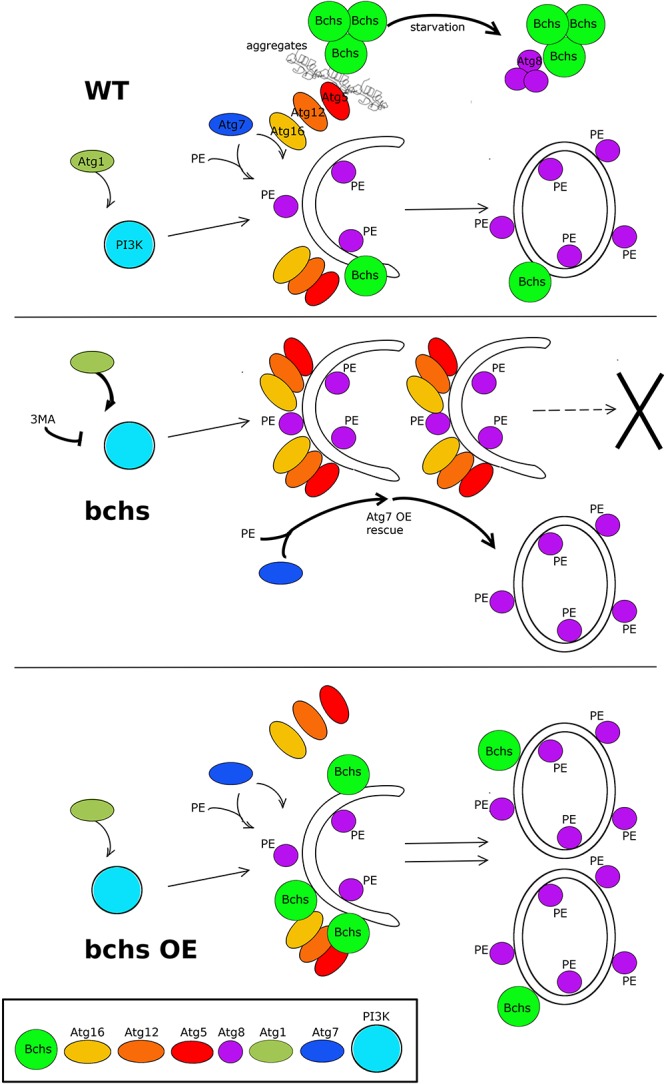
Schematic model of the possible relationship of Bchs and the autophagy pathway in the clearance of aggregated proteins and Atg8-carrying vesicle production. In the wild type situation (top), Bchs associates preferentially with Atg5 during the induction of aggrephagy (squiggly lines), and this association is suppressed by starvation-induced autophagy, wherein Bchs colocalizes more with Atg8. In the *bchs* case (middle), full maturation to Atg8-marked vesicles is reduced (—>X), and this is epistatic to upstream PI3K activation or inhibition by Atg1 or 3MA, but can be overcome by over-expressing Atg7 (thick arrows). Bchs over-expression (bchs OE; bottom) drives the maturation of autophagic vesicles to the Atg8-marked stage.

Epistatic relationships with *Atg7* are strikingly similar to the aforementioned with autophagy inhibitors. *Atg7* loss by itself phenocopies *bchs*, but only exacerbates weaker *bchs* alleles. Conversely, overexpression of Atg7, unlike rapamycin, is able to rescue even the loss of all BEACH domain Bchs isoforms. This would be surprising if Bchs were a strictly selective autophagy receptor for aggrephagy, and argues that a general boost in autophagy via Atg7 suffices to ameliorate neuronal death. These pieces of evidence suggest that Bchs acts upstream of Atg7 in the same pathway, and that the block in autophagy can be overcome by Atg7, although Atg7’s function in conjugation of the Atg12-Atg5-Atg16 complex is nominally upstream of the step toward mature lipidated Atg8-carrying vesicles (schematized in Figure 8).

The accumulation of Atg5 compartments in *bchs* mutants, but a greatly increased number and intensity of Atg8-carrying vesicles after Bchs over-expression (Figure 4), indicate that Bchs may be involved in the progression from earlier steps of autophagy, wherein it associates with Atg5-positive phagophores during the induction of aggrephagy, to later Atg8-involving steps (schematized in Figure 8, top panel, left). Such a role for Bchs is also consistent with the greatly increased quantity of p62/Ref(2)p marker in *bchs* mutants, which we documented in an earlier publication (also see Supplementary Figure S1D; Hebbar et al., 2015).

Interestingly, and contrary to expectations based on earlier publications proposing a strictly aggrephagy-related role for Bchs (Filimonenko et al., 2010), we observe low overall colocalization in resting neurons with autophagy machinery in general (Rr is on the order of 0.2–0.25 for Atg5 and Atg8), and even anti-localization of the shorter 2nd and 3rd isoforms (see Figure 1). Moreover, the autophagosome-colocalizing population of Bchs associates with different vesicle populations, dependent on the autophagy-inducing agent. Our results support a scenario whereby aggregating proteins–Htt polyQ in this case– increase Bchs’ association with (presumably early) Atg5-compartments, whereas starvation suppresses Atg5 localization in favor of mature Atg8 compartments. As indicated above, these observations may point to multiple Bchs functions, and different factors contributing to neuronal degeneration in addition to a strict role of aggrephagy (Lim and Kraut, 2009).

Low overall colocalization of Bchs with early and late autophagosomes contrasts with the high colocalization (Rr of ∼0.7) with Rab11, which is reduced by various forms of autophagic induction (see Figure 7). Bchs and Rab11 are postulated to perform antagonistic roles (Khodosh et al., 2006; Lim and Kraut, 2009), with Bchs promoting synaptogenesis while Rab11 inhibits it. Rab11 may antagonize Bchs through competitive routing of autophagosomal membrane sources under different autophagy stimuli (Longatti et al., 2012; Puri et al., 2013), for example under fed vs. starv conditions, although a change in rab11 vesicle number, size, or intensity was not apparent after autophagy induction (data not shown).

A previous study found Bchs enriched in vesicles at synaptic boutons of larval axon terminals (Khodosh et al., 2006). Notably, the initiation of autophagosome biogenesis occurs distally and constitutively in the neurite tips of mouse primary neurons and these then mature as they are transported retrograde in a dynein-dependent manner toward the cell soma (Maday et al., 2012). Further, fusion between autophagosomes and late endosomes at neuronal termini is required for recruitment of the necessary motor proteins for retrograde transport of the fused compartment, the amphisome (Cheng et al., 2015). Given the role of BEACH domain proteins in vesicle fusion events, and Bchs’ effects on transport of endolysosomes in motor neuron axons (Lim and Kraut, 2009), involvement in this early step of autophagic clearance in synaptic termini (Friedman et al., 2012) seems a likely scenario for its function.

A number of exocytic SNARE proteins are required at the step of autophagosome biogenesis to promote Atg9 recycling through tubulo-vesicular clustering and membrane fusion at phagophore assembly sites (Nair et al., 2011). In addition, the Q-SNARE Syx17 mediates the fusion of autophagosomes with late endosomes and lysosomes in mammalian cells and *Drosophila* (Itakura et al., 2012; Takáts et al., 2013). Intriguingly, the BEACH protein LYST binds to SNARE proteins (Tchernev et al., 2002), and we also detected Bchs-dependent changes in Syx17 distribution in neurons, raising the possibility of Bchs interacting with this or other SNAREs to promote vesicle fusion. On the other hand, our finding of increased numbers of Atg8 vesicles after over-expression of the large BEACH isoform is reminiscent of the increased numbers of lysosomes that were seen after LYST over-expression (Durchfort et al., 2012). This would be consistent with a similar role in vesicle fission (not fusion), as LYST and related proteins are thought to perform (Kypri et al., 2013), whereby BEACH proteins are implicated as accessories in both fission and fusion events of different vesicle populations (Cullinane et al., 2013). Bchs’ possible role in vesicle trafficking and membrane dynamics, in the context of its recently reported genetic interactions with sphingolipid lipases (Hebbar et al., 2015) will be an interesting avenue to investigate in the future.

## Materials and Methods

### *Drosophila* Stocks and Maintenance

Flies were raised on standard yeast/cornmeal agar food at 25°C. The fly lines used were *bchs58(O)*, *bchs58(M)*, *bchs17(M)*, *Df(2L)clot7*, *yw; EPgy2 Atg7[EY10058]* (Bloomington Drosophila Stock Center); *w; Atg7[d77]/CyO-GFP*, and *yw; UAS-mCherry-Atg8a* and UAS-Atg1(6A) were kindly provided by Thomas Neufeld. *yw;*+*; UAS-RFP-Atg5*, *w;*+; *UAS-Rab11-GFP*, and the Htt-polyQ expressing lines *w; UAS-Htt exon1-Q20* and *-Q93* were generous gifts of Katja Köhler, Sean Sweeney, Henry Chang, and Larry Marsh, respectively. The *bchsLL03462* and UAS-Atg1^*GS10797*^ stocks were obtained from Drosophila Genomics and Genetic Resources Stock Center in Kyoto Institute of Technology. *bchs* RNAi line #45028 was from the Vienna Drosophila Resource Center (VDRC). The eve-Gal4>UAS-CD8-GFP stock (an *even-skipped* driver combined with a membrane-bound GFP marker) was a kind gift of Miki Fujioka.

### RT-PCR of *bchs* and *atg7* Transcripts and Generation of GFP-Bchs

Larval or adult brains were homogenized in 400 μL of TRIzol^®^ reagent (Life Technologies, 15596-026), and total RNA was extracted with chloroform:TRIzol^®^ 1:5. RT PCR was performed using Promega (A5000) M-MLV reverse transcriptase as per manufacturer’s instructions, and PCR was carried out on each cDNA sample with GoTaq^®^ polymerase (Promega, M3005). Gene specific primers used to amplify *bchs* splice isoform 2 were forward = GCAAACAGTTCAGACAATATAC and reverse = AAGATCCTTTATCAGCTGCTTGGC, and splice isoform 3 were forward = GATGGACAGAAAACGATGCTACC and reverse = TTCGCAGGATGAATTTCTCGTG. *atg7* specific primers were: forward = TCCGCAGACGGATTGATCTC and reverse = TGAACATCGATGACAGCCTTG; PCR primers for *rp49* were: forward = AGTCGGATCGATATGCTAAG and reverse = AGTAAACGCGGTTCTGCATG. *bchs* isoforms were cloned into pUAST vector containing a GFP tag at the 5′ end. Transgenic animals were generated by Best Gene (Chino Hills, CA, United States).

### Motor Neuron Viability Assay

Third instar larvae were dissected, fixed 30 min in 4% paraformaldehyde (Sigma Aldrich, P6148), blocked 1 h in 5% bovine serum albumin (BSA) (PAA Laboratories, K41-001) in 0.1% Triton X-100 PBS (PBT), and incubated overnight at 4°C with primary antibodies: 1:500 anti-GFP (Clontech Laboratories, 632377), 1:10 1D4 anti-fasciclin II and 1:20 22C10 anti-futsch (Developmental Studies Hybridoma Bank), and washed in PBT. Secondary antibodies were 1:800 Cy2-conjugated goat anti-rabbit and 1:800 Cy3-conjugated goat anti-mouse (Jackson ImmunoResearch Laboratories, 111-225-144 and 115-165-146) in PBT. Motor neuron viability was scored by inspection. RP2 survival was scored as a percentage over total hemisegments and repeated in triplicate. Chi-square statistical test was performed.

### Drug Treatments and Western Analysis

Embryos were collected every 3 h on apple juice agar plates, and incubated at 25°C for 24 h; hatching first instar larvae were transferred into 2.0 mL Eppendorf tubes with food containing 0.05% (v/v) ethanol (vehicle control), 1 μM rapamycin (AG Scientific), 0.2 or 2 μM Wortmannin (AG Scientific; R-1018) or 5 mM 3-methyladenine (Calbioche^®^ m, Merck Millipore, 189490), and dissected as third instar larvae. 10 μM Chloroquine was added to primary cultures for 2 h before fixation and staining with anti-mCherry. For Westerns, 5 third instar larval brains or 3 heads per treatment per lane were collected, frozen on dry ice, and ground with a pestle in RIPA lysis buffer (Thermo Fisher Scientific) with protease inhibitor cocktail. Rabbit anti-Bchs [for details see Lim and Kraut (2009)] or Rabbit anti-pS6K (Cell Signaling) were diluted 1:6000 and 1:1000, respectively, incubated on blots overnight at 4C, and detected with HRP-coupled secondary and SuperSignal West Pico plus reagent (Thermo Fisher Scientific).

### Quantitative Size Analysis of Ubiquitinated Aggregates in Larval Neuromuscular Junctions

Aggregates were detected with 1:1000 mouse anti-poly-ubiquitin (clone FK2, Enzo Life Sciences, BML-PW8810) and 1:800 Cy5-goat anti-mouse and 1:500 Cy2-goat anti-horse radish peroxidase (Jackson ImmunoResearch Laboratories, 115-175-146 and 123-095-021). Image stacks were acquired under 40× objective lens with a DeltaVision OMX^®^ microscope (Applied Precision) and deconvolved with softWoRx^®^ 5.0. To measure aggregate area, ImageJ particle analysis was used (Ullrich et al., 1996). The measurement scale (in microns) was defined on the image projection, adjusted to a segmentation threshold of 70, and converted to a binary image. A region of interest (ROI) was marked and ‘Analyze Particles’ used to measure the area of particles within the ROI. Particle size was categorized into 0–1, 1.1–10, and 10.1–50 μm^2^. Percentages of neuromuscular junctions with aggregates in each of the three groups were calculated for each experimental set, which was repeated in triplicate, and a Chi-square statistical test was performed.

### Primary Neuron Culture From Third Instar Larval Brains, and Imaging of GFP-Bchs-1

The protocol was modified from Kraft et al. (2006). Larvae were washed with 90% ethanol three times and sterile water twice to remove debris and contaminants. Four third instar larval brains were extracted in Shields and Sang, bacto-peptone and yeast extract (Sigma-Aldrich, S8398) medium containing penicillin-streptomycin and antibiotics-antimycotics (PAA Laboratories, P11-002), washed with sterile hemolymph-like 3 (HL-3) saline (70 mM NaCl, 115 mM sucrose, 5 mM trehalose, 5 mM KCl, 20 mM MgCl_2_, 1 mM CaCl_2_, 10 mM NaHCO_3_, 5 mM HEPES, pH 7.4) three times, and incubated with 0.5 mg/mL collagenase type 1 (Sigma-Aldrich, C1639) in HL-3 1 h at room temperature. Brain tissue was then washed three times with complete medium with 10% heat-inactivated fetal bovine serum (HyClone, Thermo Fisher Scientific, SH30070.03), 20 μg/mL of bovine pancreas insulin (Sigma-Aldrich, I1882), penicillin-streptomycin and antibiotics-antimycotics. Brains were dissociated into a final volume of 150 μL as a single cell suspension by trituration and then aliquoted onto 22 mm^2^ acid-washed cover-slips coated with 30 μg/mL of mouse laminin (BD Biosciences, 354232) and 167 μg/mL of concanavalin A (Sigma Aldrich, C0412) in 35 mm culture dishes and allowed to attach ∼12 h. 900 μL of complete medium was added to the culture dish and incubated for 1–4 days.

For the colocalization in Figure 1, live primary neurons from larval offspring of elav-Gal4>mCherry-Atg8a or elav-Gal4>UAS-RFP-Atg5 crossed with UAS-GFP-bchs-1, 2, or 3 were imaged at 60× magnification on the DeltaVision microscope as above, using Z-stack acquisition of 200 nm optical slices with interleaving green and red channels at 150 ms exposures. 100 image stacks for each colocalization were examined at each Z-level, and where vesicular localization of the Bchs isoform was detectable, the individual vesicle was then visually inspected in red/green image pairs for complete, non-, or partial overlap of Bchs with mCherry-Atg8a or RFP-Atg5. The colocalization algorithm used below was not applied here because of a high cytoplasmic expression level of the GFP-Bchs isoforms. Each vesicle expressing Bchs was only counted once. Standard error was calculated by averaging the percentages in each of the three categories (“yes,” “no,” or “partial”) over the data separated into three equal batches of images.

### Immunocytochemistry and Image Analysis of Compartments in *bchs* Primary Neurons

*bchs* and wild-type primary neurons were fixed with 4% paraformaldehyde in PBS for 15 min at room temperature and blocked with 5% normal goat serum (Life Technologies, 50-062Z) in 0.05% Triton X-100 PBS for 1 h. Antibody staining was performed as above with 1:1000 mouse anti-poly-ubiquitin (clone FK2, Enzo Life Sciences, BML-PW8810) and 1:500 rabbit anti-drAtg5 (Novus Biologicals, NB110-74818) or 1:500 rabbit anti-drAtg8a (a kind gift of Katja Köhler); anti-p62 (1:500) was kindly donated by Nezis et al. (2008). Samples were incubated with 1 μg/mL of 4′,6-diamidino-2-phenylindole (DAPI), dihydrochloride (Life Technologies, D1306) in PBS for 3 min, washed briefly with PBS, then water, and imaged at 60× magnification.

For the image analysis in Figure 4, images of primary neuron cultures were acquired as Z-stacks, with the step of 200 nm in between slices, using the same exposure, light intensity and filter settings for every condition. Collected stacks were then deconvolved as above, and converted into 16-bit TIFF stacks in FIJI^1^ using LOCI BioFormats plug-in (Linkert et al., 2010). Each stack was then Z-projected using the maximum intensity method. Images were inspected under DAPI channel and only single cells, or small aggregates (<5 cells) with clearly separated cell nuclei were used for further spot counting. Because of varying spot intensity relative to cell body, and variable background signal, spots were counted manually, after setting the same display range of values. The cell number N used for calculations varied between conditions (N_average_ ∼100), but was not less than N_min_ = 50. The average spot number per cell and the standard error of the mean (SEM) were calculated. To measure spot characteristics, projected images were masked using the Maximum Entropy threshold method (Kapur et al., 1985). Brightness and size distribution and SEM of selected spots were measured using the Analyze Particles FIJI command.

Analysis of mCherry-Atg8 and Syx17 compartment size and brightness for Supplementary Figure S8 was carried out on images acquired with a Yokogawa Spinning Disk confocal microscope with a 100×/1.4 NA Silicon immersion lens, using the Fiji plugin “find maxima” with the same manual threshold for all images, such that obvious spots of expression could be detected. All images were collected with identical exposure and laser power settings for the mCherry and Syx17 channels, and controls were processed in parallel for all experiments. For each condition, 80–100 individual images each with several neurons were assessed.

### Quantitative Colocalization Analysis of Bchs With Different Compartmental Markers

Autophagy was induced in primary neurons by 5 h of nutrient starvation with HL-3, 5 h of 50 nM rapamycin in complete medium or Huntingtin Q93 expression. Controls (basal autophagy) were incubated in complete medium. Antibody staining was performed as above. For mCherry-Atg8a or RFP-Atg5, primary antibodies were 1:500 rabbit anti-Bchs and 1:100 mouse anti-DsRed (BD Pharmingen^*TM*^, 551814). For Rab11-GFP, primary antibodies were rabbit anti-Bchs and 1:1000 mouse anti-GFP (Clontech Laboratories, 632375). Colocalization analysis was done with the ImageJ plugin ‘Intensity Correlation Analysis’ after channel splitting and background subtraction (Li et al., 2004). Rr (Pearson’s correlation coefficient), Ch1:Ch2 ratios, M1 and M2 (Manders’ colocalization coefficient for channel 1 and 2, with thresholding) were tabulated for each image. Unpaired Student’s *t*-test was used to calculate *p*-values between treated and control groups.

### Time-Lapse Imaging of GFP-Bchs With RFP-Atg5 or mCherry-Atg8a in Primary Neurons

Primary neurons expressing *GFP-Bchs* and *RFP-Atg5* or *mCherry-Atg8a* transgenes via elav-Gal4 were cultured on 35 mm glass bottom dishes (World Precision Instruments, FD35-100) coated as described above. For basal autophagy, cells were incubated in complete culture medium at room temperature and single focal-plane images were acquired for GFP-Bchs: green channel = 50% transmission, 800 ms; RFP-Atg5: red channel = 50% transmission, 800 ms; and mCherry-Atg8a: red channel = 32% transmission, 600 ms, every 10 min over 4 h, and deconvolved. For nutrient starvation, neurons were incubated in HL-3 saline at room temperature and immediately imaged as described. For transfection of Huntingtin 15Q or 128Q, 3 μL of FuGENE^®^ HD transfection reagent (Promega, E2311) was used to couple 1 μg of pCINeoHtt1955.15Q.wt or pCINeoHtt1955.128Q.wt plasmid DNA (kind gift of Anat Yanai and Mahmoud Pouladi), respectively in a 3:1 ratio in a final volume of 50 μL complete culture medium without penicillin/streptomycin and antibiotics/antimycotics for 10 min at room temperature, and added to the cells with 1 mL of complete culture medium without penicillin/streptomycin and antibiotics/antimycotics for 48 h (without medium change).

## Author Contributions

JS, KO, and RK carried out experiments, interpreted results, and prepared figures. IAG carried out the experiments. AM carried out image analysis. JS drafted the manuscript and RK revised the manuscript.

## Conflict of Interest Statement

The authors declare that the research was conducted in the absence of any commercial or financial relationships that could be construed as a potential conflict of interest.

## Footnotes

1 http://fiji.sc
