# Supplementary material for: The BEACH Domain Is Critical for Blue Cheese Function in a Spatial and Epistatic Autophagy Hierarchy
Source: Front Cell Dev Biol. 2019 Aug 2;7:129. doi: 10.3389/fcell.2019.00129 (PMC6688705; doi:10.3389/fcell.2019.00129)
Supplement: Supplementary file 1 [file Table_1.doc]

**Supplementary Figure Legends**

**BEACH domain adaptor *blue cheese* drives autophagic proliferation in Drosophila primary neurons and shuttles between vesicle populations dependent on the autophagic impetus**

Joan Sim^1^, Kathleen A. Osborne^1^, Irene Argudo Garcia^1,2^, Artur S. Matysik^1,3^, and Rachel Kraut^1,4^

**Supplementary Figure S1.** **(A)** Autophagy-enhancing or -suppressing drugs change relative strength of phosphorylated S6K (pS6K), an effector of the mTOR pathway, in Westerns of larval brain. Left-most arrowhead in A points to lower (~70kDa) band that is increased with rapamycin at 1 or 10 μM. An upper band (~100kDa) is increased in DMSO on the same blot. Wortmannin (WM) and 3-Methyladenine (3MA) appear to have roughly equal amounts of both bands, but total amounts are slightly increased in 3MA, consistent with it having a suppressive effect on autophagy.

**(B)** Larval muscles of wild type larvae fed with rapamycin show enhanced autophagosome formation, while Wortmannin-fed animals show reduced autophagy, indicated by Atg8a-mCherry. Fillets of wild-type larvae over-expressing mCherry-Atg8a in muscles via 24B-mef2-Gal4 were fed with 1 μM rapamycin or 1.5 μM Wortmannin from first instar to third instar stage. Graph represents average numbers (n=5) of punctae, +/- SEM, identified by a “find maxima” plug-in in ImageJ, with intensity cut-off at 50. Scalebar = 20 μm.

**(C)** Westerns of adult heads from flies over-expressing Atg1 RNAi, Raptor RNAi, or the Atg7 EY10058 UAS insertion line via elav-Gal4, vs. *yw/*elav-Gal4 control heads. Arrowhead points to lower (~70kDa) band that is increased in Atg7 over-expressing heads, similar to its appearance in rapamycin-fed larval brain (compare to left-most lane in first blot in A).

**(D)** Quantification of average Atg8 or p62 spot numbers per cell in primary cultured larval neurons from *bchs17(M)* or *yw* (WT) controls treated with 50nM rapamycin (‘rapa’, dark green bars) or not (‘no rapa’, light green bars), as in Fig. 5. Quantification was carried out as in Fig. 4, and described in Methods section. Error bars represent S.E.M.

**Supplementary Figure S2.** (Corresponds to Fig. 4.) Primary neurons prepared from *bchsLL03462/cl7* larval brain, and the same genotype in the GFP-bchs-1 rescue background, labelled for **(A)** Atg5 and **(B)** Atg8, also stained with DAPI (blue) to show cell locations and anti-poly-ubiquitin (green). Scalebar =10 µm.

**Supplementary Figure S3.** Montage of individual neurons in primary culture from wild type (+/+) vs. the strong allelic combination *bchsLL03462/Df*, alone or rescued by expression of the GFP-bchs-1 transgene. Images shown here of anti-Atg5 labelled neurons (detected in the red channel) were used for the analysis in Fig. 4A, B.

**Supplementary Figure S4.** **(A)** Montage of individual neurons in primary culture from the allelic combination *bchs17(M)/Df*, and *bchs58(M)/Df*, each alone or rescued by expression of the GFP-bchs-1 transgene. Images shown here of anti-Atg5 neurons (detected in the red channel) were used for the analysis in Fig. 4A, B. **(B)** Primary neurons cultured from third instar larval brains of the indicated genotypes, aged 4 days, and labelled with DAPI to show cell location, anti-poly-ubiquitin, and Atg5. The top-right inset is a 5x digital zoom for the indicated region of interest by an arrow showing the deposition of ubiquitinated aggregates. Scalebar =10 µm.

**Supplementary Figure S5.** Montage of individual neurons in primary culture from wild type (+/+) vs. the strong allelic combination *bchsLL03462/Df*, alone or rescued by expression of the GFP-bchs-1 transgene. Images shown here of anti-Atg8 neurons (detected in the red channel) were used for the analysis in Fig. 4E, F.

**Supplementary Figure S6.** **(A)** Montage of individual neurons in primary culture from the allelic combinations *bchs17(M)/Df* and *bchs58(M)/Df*, each alone or rescued by expression of the GFP-bchs-1 transgene. Images shown here of anti-Atg8 neurons (detected in the red channel) were used for the analysis in Fig. 4E, F. **(B)** Primary neurons cultured from third instar larval brains of the indicated genotypes, aged 4 days, and labelled with DAPI to show cell location, anti-poly-ubiquitin, and Atg8. The top-right inset is a 5x digital zoom for the indicated region of interest by an arrow showing the deposition of ubiquitinated aggregates. Scalebar =10 µm.

**Supplementary Figure S7**. Htt Q93-induced ubiquitinated aggregates colocalize with autophagic vesicles to the same extent in *bchs* and wild type (+/+) primary neurons. Neurons from third instar larval brains of the indicated genetic backgrounds, and overexpressing the HttQ93 transgene via elav*-*Gal4 were immunostained for Atg5 **(A)** or Atg8 **(B)** and anti-poly-ubiquitin. Single-plane images were acquired at 100x magnification. Scalebar = 10 µm. Pearson's correlation coefficient R between poly-ubiquitin and either Atg8 or Atg5 were calculated from >50 images.

**Supplementary Figure S8.** **(A-F)** Control mCherry-Atg8-expressing *yw* (+/+) or *bchs* RNAi (VDRC #45028)-expressing primary neurons from third instar larval brains grown for 3 days and exposed to Chloroquine 10uM or vehicle for 2h prior to fixation and staining with anti-mCherry and DAPI. mcherry-Atg8 signal in 80-100 images (typically with 3-6 neurons/image) was analyzed per condition and quantified using Fiji “find maxima” and an in-house developed script to detect and quantify regions, outlined in yellow in right-hand panels. Significance stars are as in main text.

**(G-L)** Control *yw* (+/+), or *bchs*17(M) and bchs58(M), with or without GFP-bchs-1 expressed in the background via elav-Gal4, stained for the Snare protein Syx17. **(J)** shows quantification of Syx17, as in **(E, F)**.

**Supplementary Figure S9.** Live imaging of GFP-Bchs with RFP-Atg5 and mCherry-Atg8a during aggrephagy and starvation. Primary neurons cultured from third instar larval brains expressing GFP-bchs-1 and RFP-Atg5 **(A, B, C, D)** or mCherry-Atg8a **(E, F, G, H)** were imaged live at time intervals of 10 minutes under the stated conditions of autophagy induction: incubation with complete medium (control), 48 hours after transfection with Htt Q15 or Htt Q128. The top-right inset shows a 5x magnification of the region of interest indicated by an arrow. Each single-plane image was acquired at 100x magnification.

**Supplementary Movie 1**. Live imaging of GFP-Bchs-1 and RFP-Atg5 in control, untreated primary neurons from larval brain.

**Supplementary Movie 2**. Live imaging of GFP-Bchs-1 and RFP-Atg5 in primary larval neurons under starvation conditions.

**Supplementary Movie 3**. Live imaging of GFP-Bchs-1 and RFP-Atg5 in primary larval neurons transfected with control construct expressing Htt Q15.

**Supplementary Movie 4**. Live imaging of GFP-Bchs-1 and RFP-Atg5 in primary larval neurons transfected with the aggregate-producing Htt Q128 construct.

**Supplementary Movie 5**. Live imaging of GFP-Bchs-1 and mCherry-Atg8 in control, untreated primary neurons from larval brain.

**Supplementary Movie 6**. Live imaging of GFP-Bchs-1 and mCherry-Atg8 in primary larval neurons under starvation conditions.

**Supplementary Movie 7**. Live imaging of GFP-Bchs-1 and mCherry-Atg8 in primary larval neurons transfected with control construct expressing Htt Q15.

**Supplementary Movie 8**. Live imaging of GFP-Bchs-1 and mCherry-Atg8 in primary larval neurons transfected with aggregate-producing Htt Q128 construct.
