# Supplementary figures and images for: The BEACH Domain Is Critical for Blue Cheese Function in a Spatial and Epistatic Autophagy Hierarchy
Source: Front Cell Dev Biol. 2019 Aug 2;7:129. doi: 10.3389/fcell.2019.00129 (PMC6688705; doi:10.3389/fcell.2019.00129)

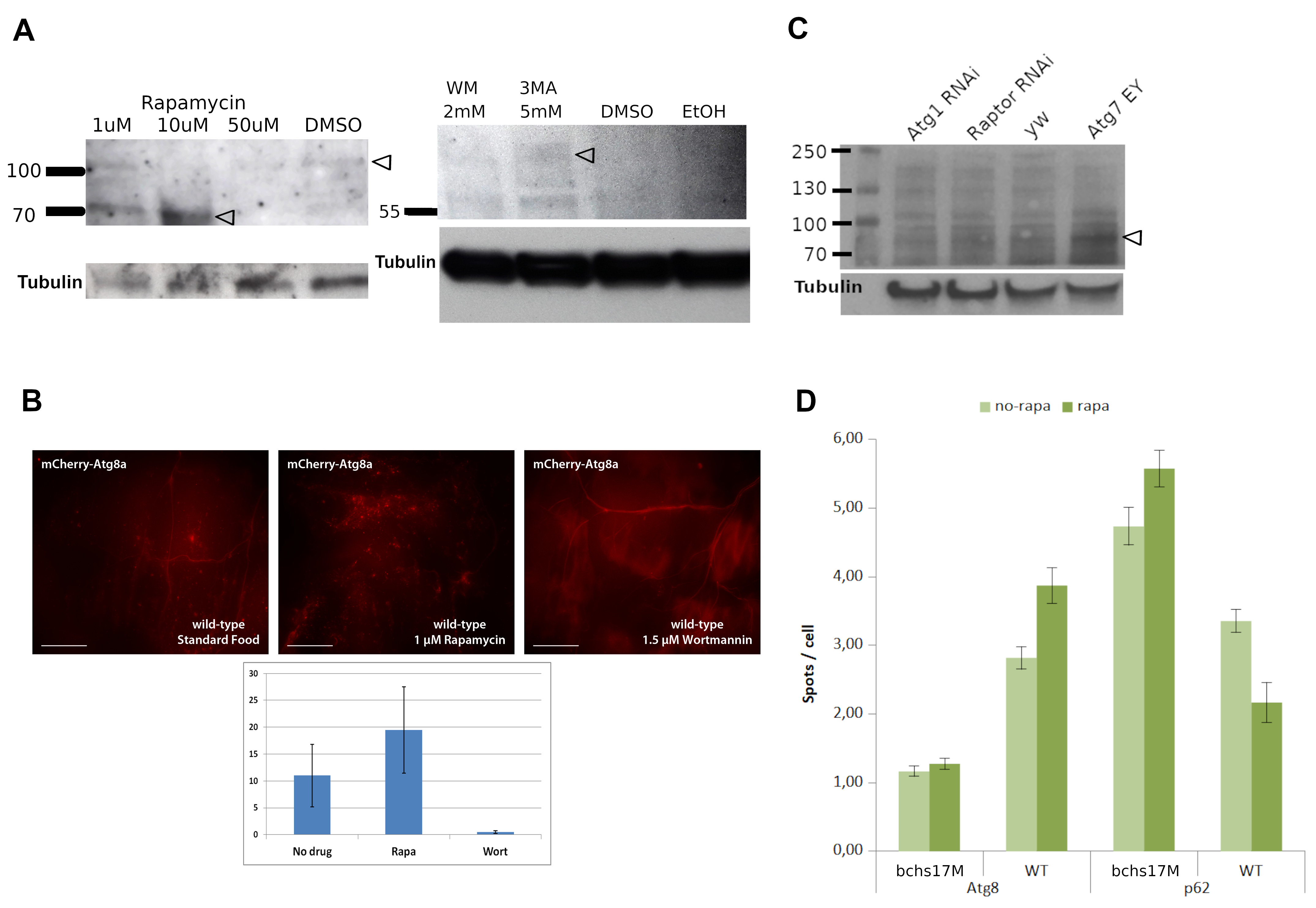

Supplement: Supplementary file 10 [file Image_1.JPEG]

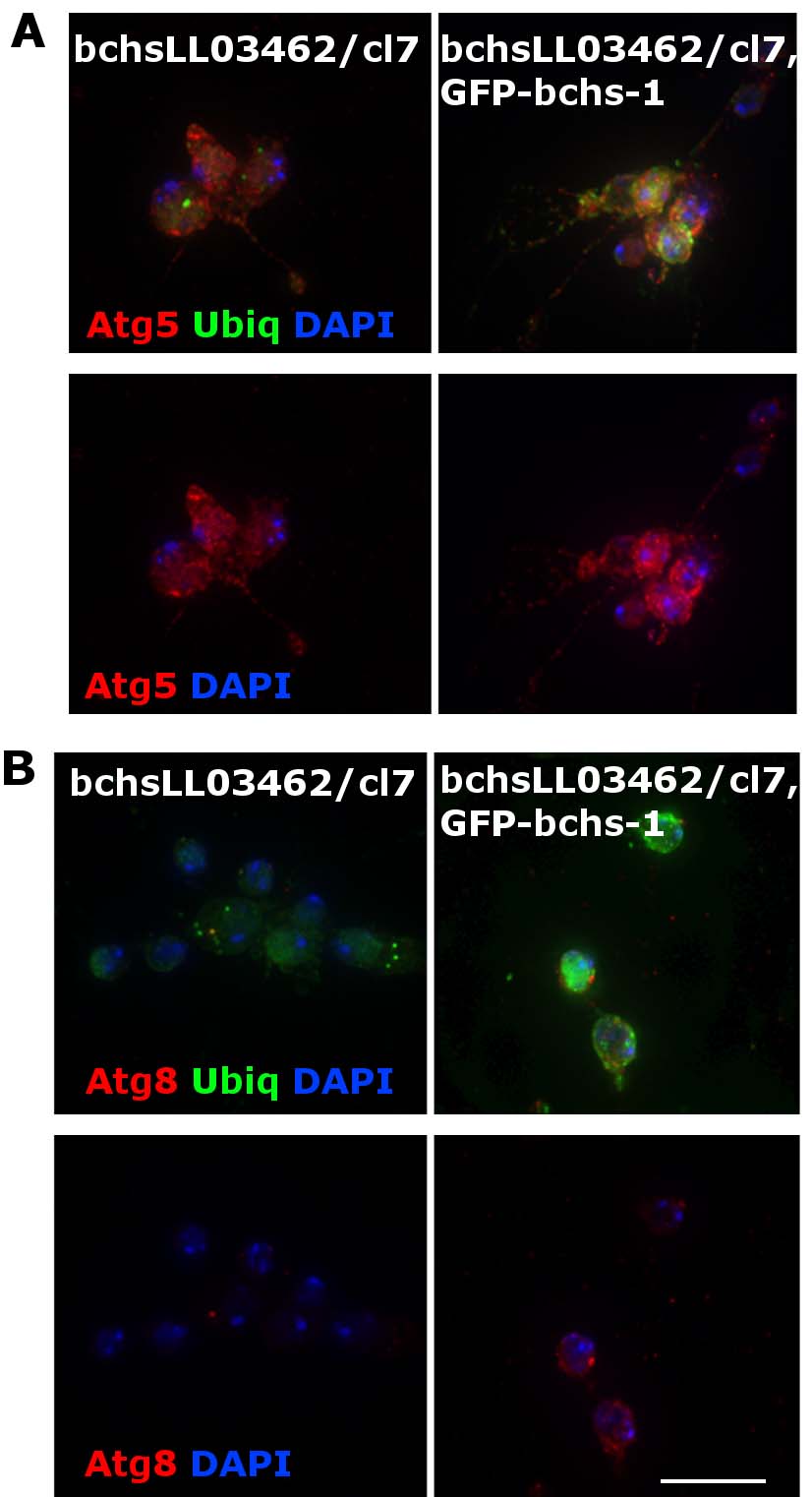

Supplement: Supplementary file 11 [file Image_2.JPEG]

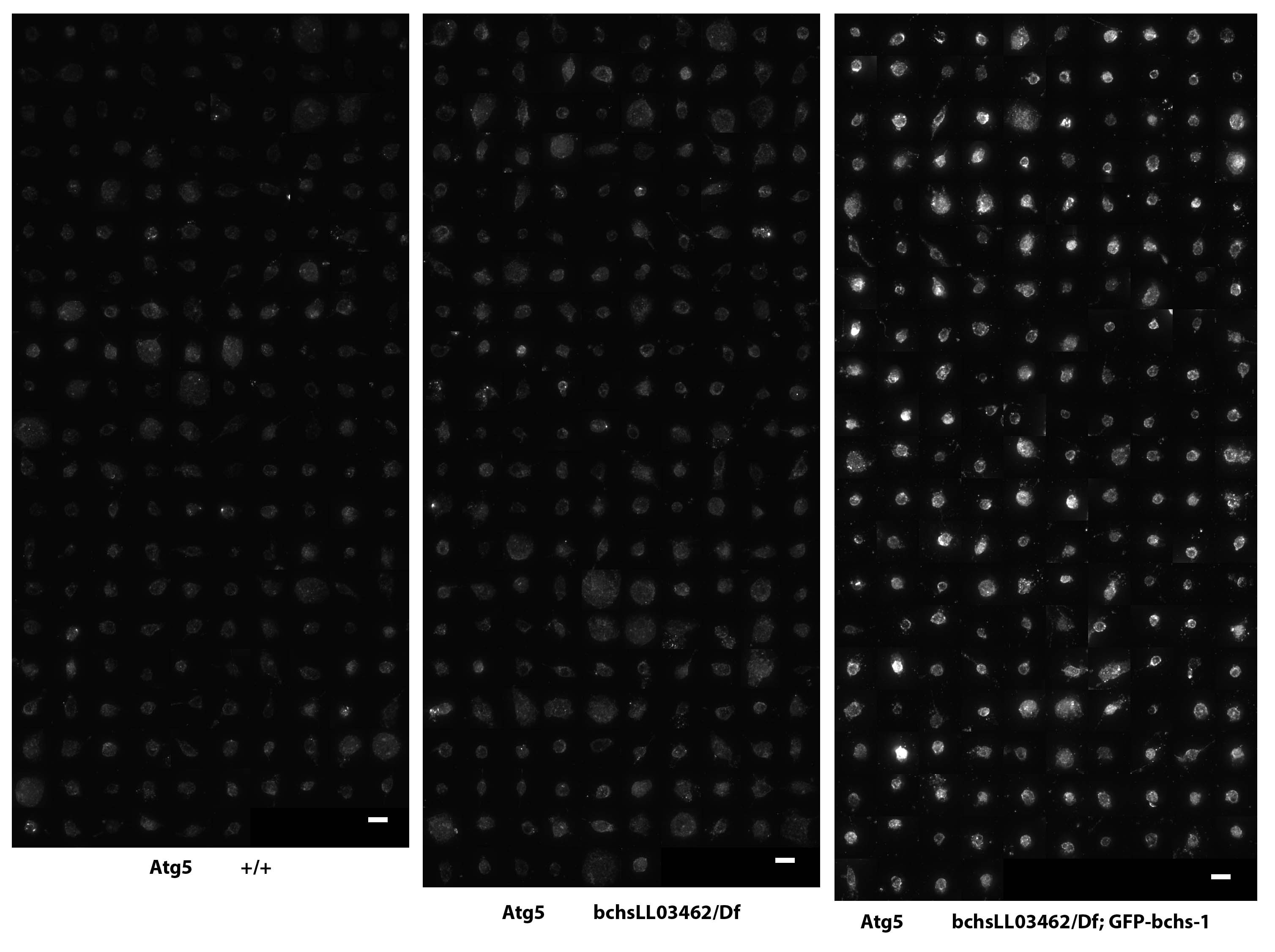

Supplement: Supplementary file 12 [file Image_3.JPEG]

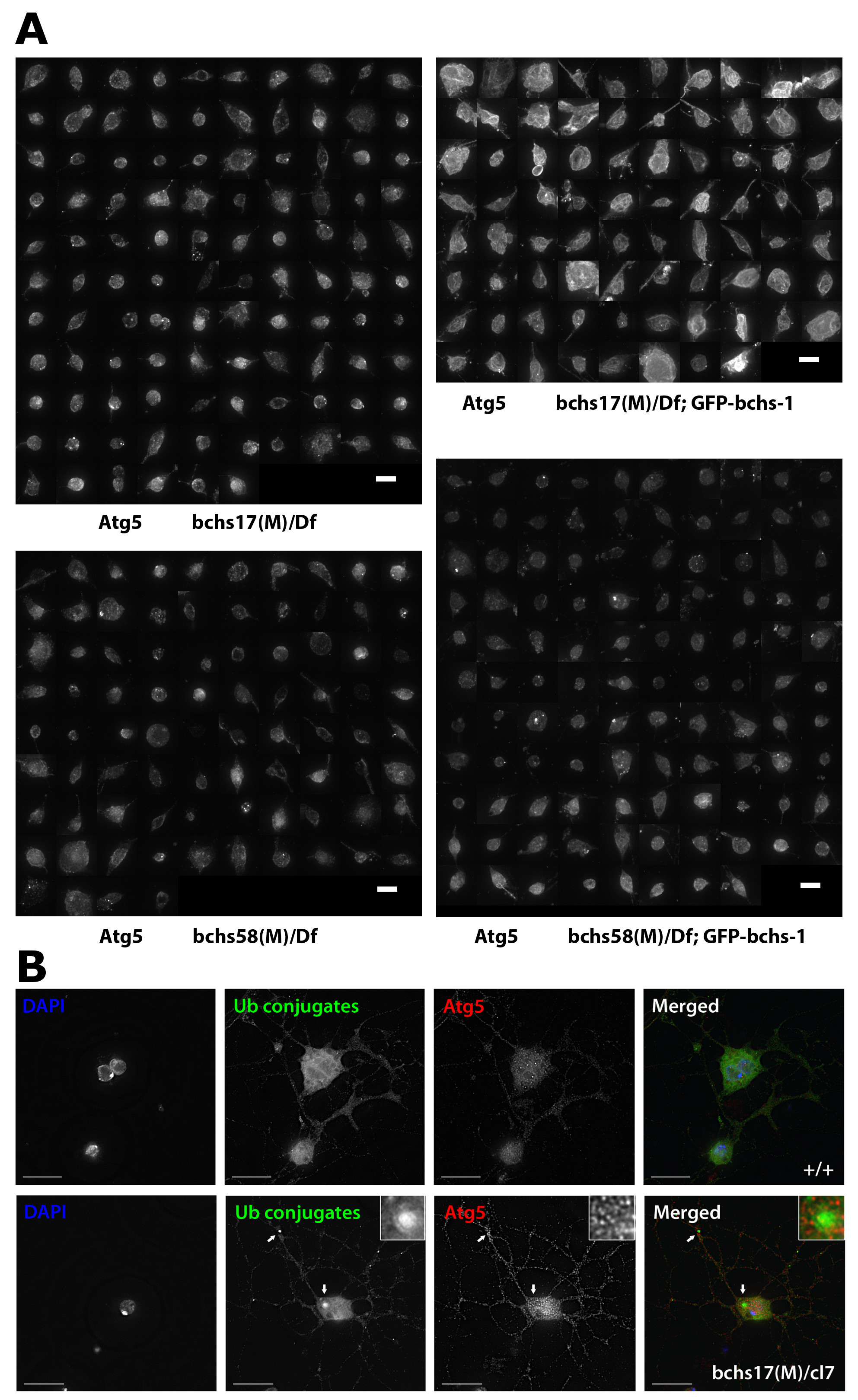

Supplement: Supplementary file 13 [file Image_4.JPEG]

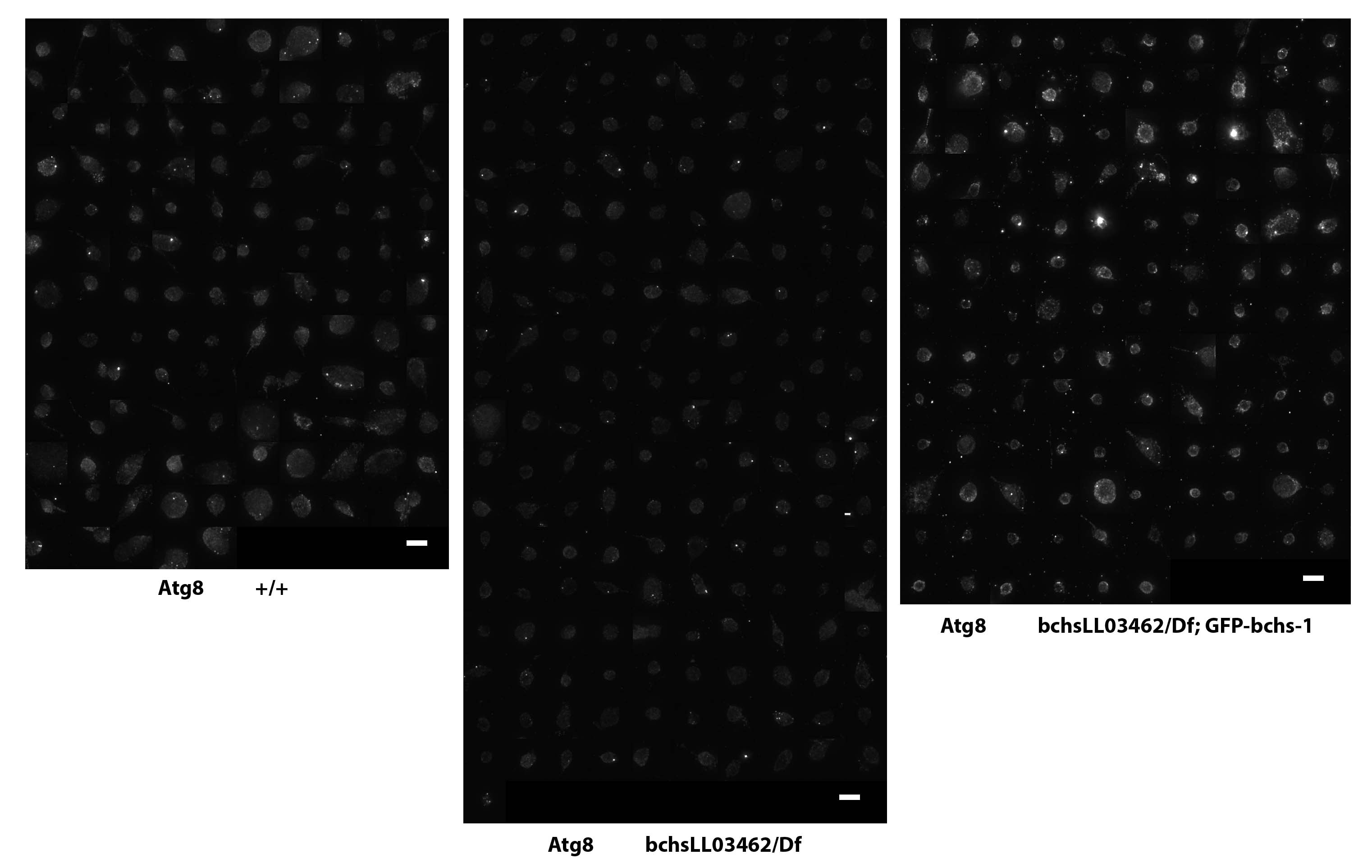

Supplement: Supplementary file 14 [file Image_5.JPEG]

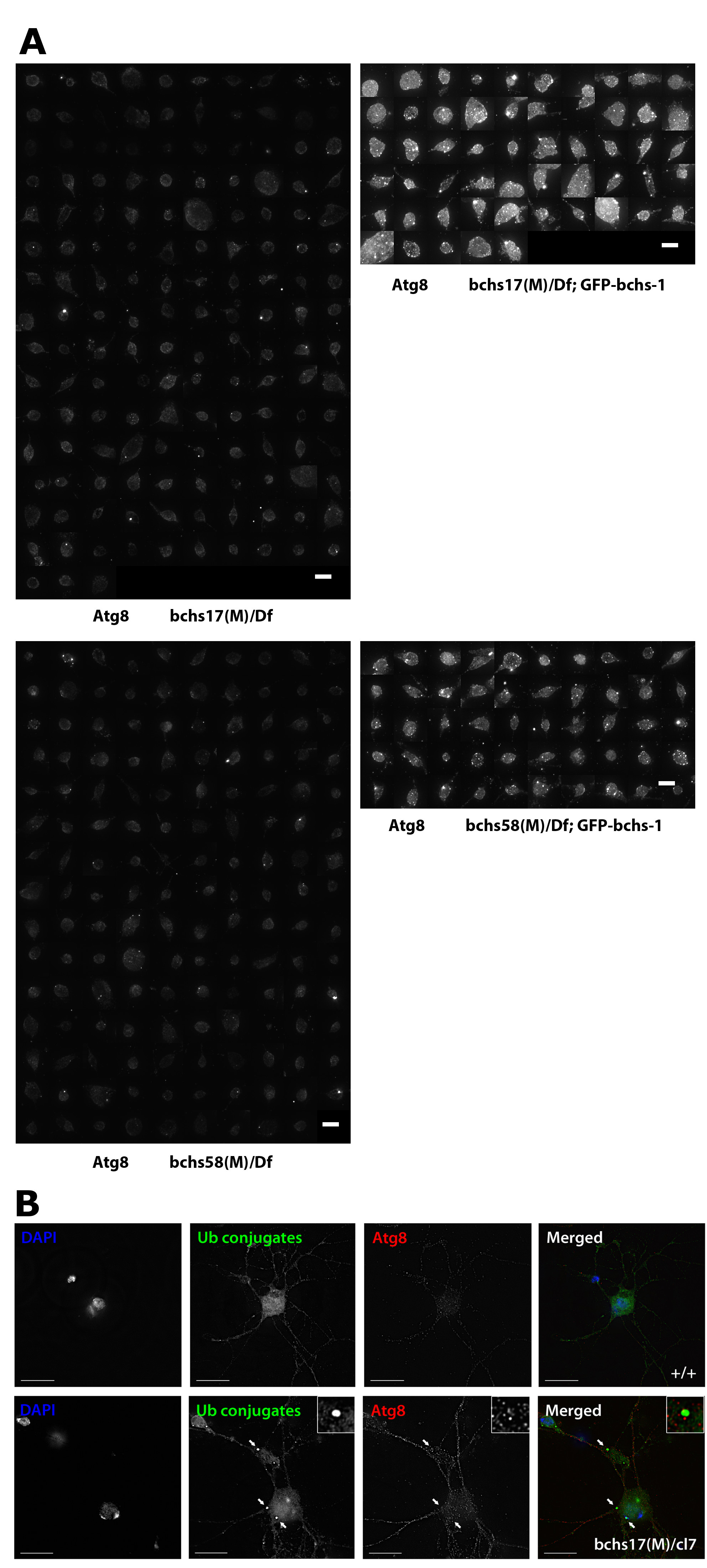

Supplement: Supplementary file 15 [file Image_6.JPEG]

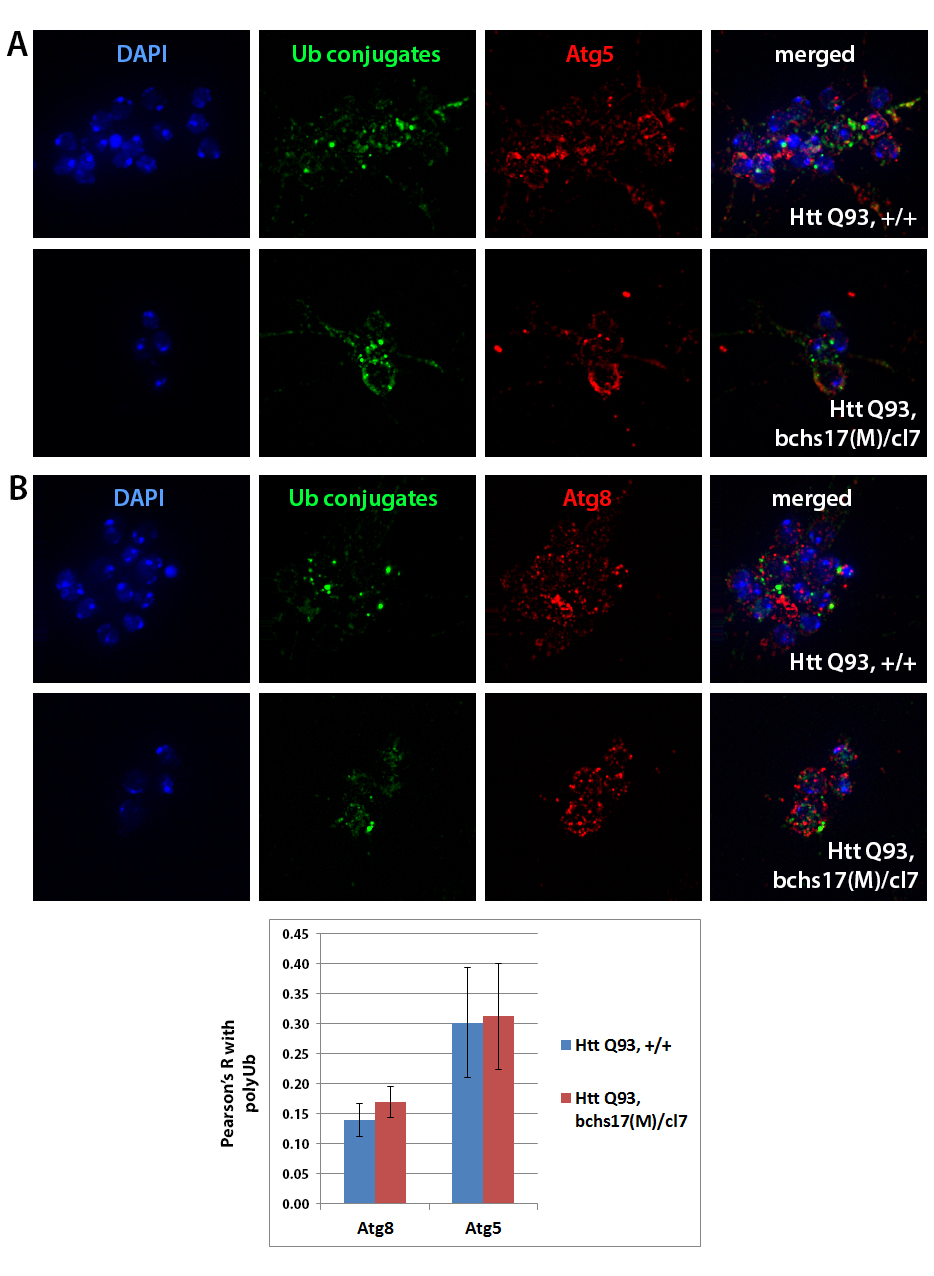

Supplement: Supplementary file 16 [file Image_7.JPEG]

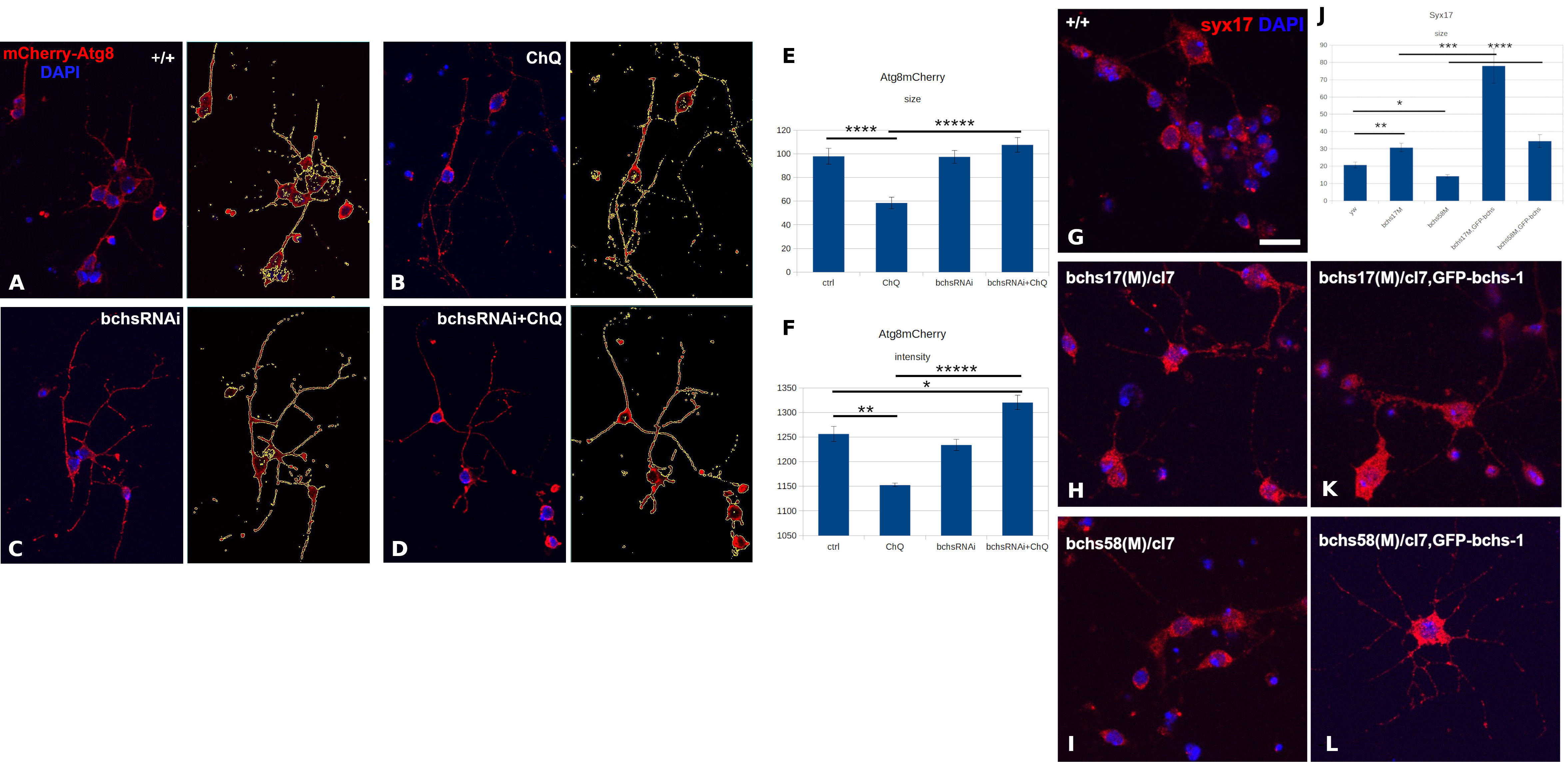

Supplement: Supplementary file 17 [file Image_8.JPEG]

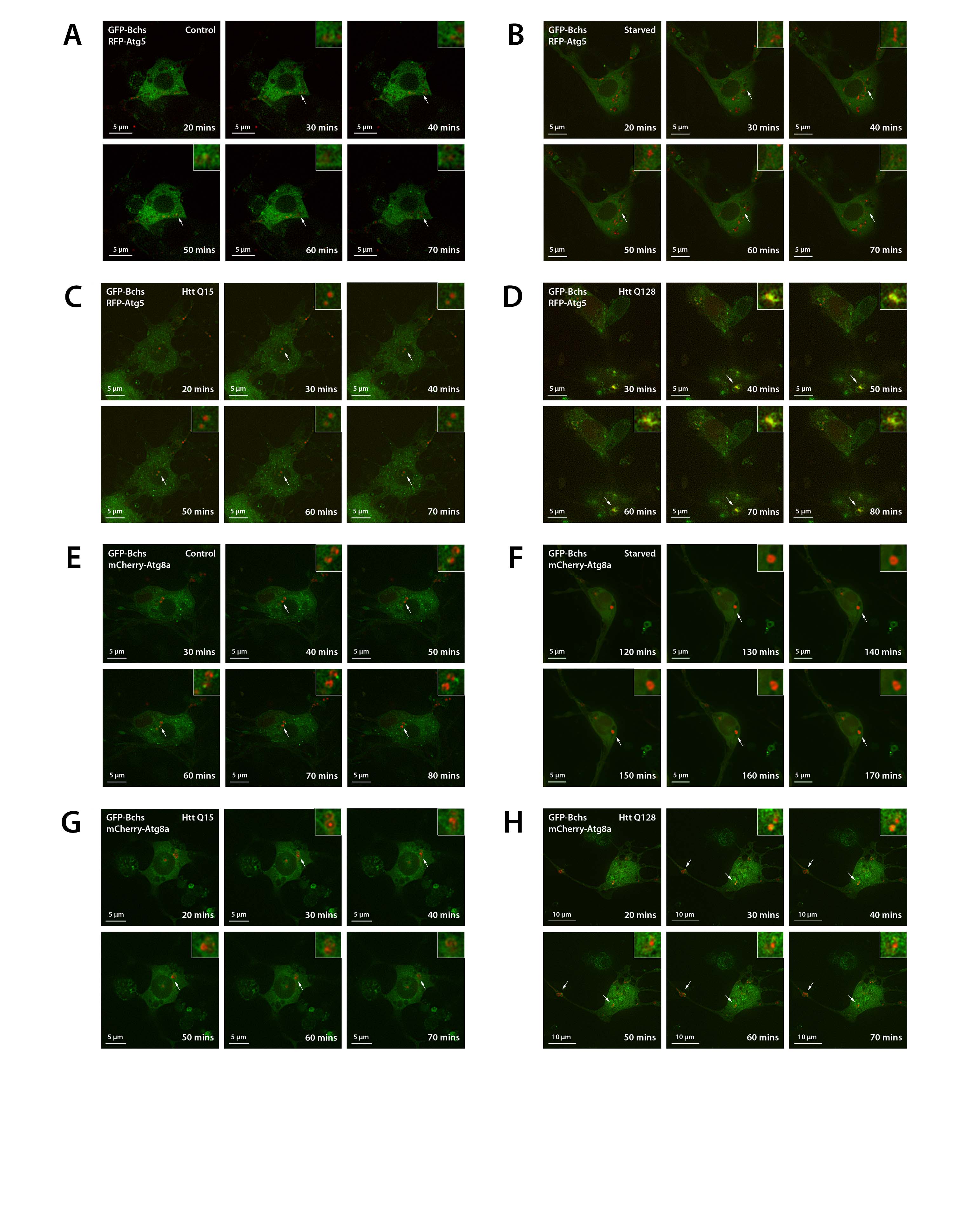

Supplement: Supplementary file 18 [file Image_9.jpeg]
